# Savanna elephant numbers are only a quarter of their expected values

**DOI:** 10.1371/journal.pone.0175942

**Published:** 2017-04-17

**Authors:** Ashley S. Robson, Morgan J. Trimble, Andrew Purdon, Kim D. Young-Overton, Stuart L. Pimm, Rudi J. van Aarde

**Affiliations:** 1 Conservation Ecology Research Unit (CERU), Department of Zoology and Entomology, University of Pretoria, Pretoria, South Africa; 2 Nicholas School of the Environment, Duke University, Durham, North Carolina, United States of America; University of Tasmania, AUSTRALIA

## Abstract

Savannas once constituted the range of many species that human encroachment has now reduced to a fraction of their former distribution. Many survive only in protected areas. Poaching reduces the savanna elephant, even where protected, likely to the detriment of savanna ecosystems. While resources go into estimating elephant populations, an ecological benchmark by which to assess counts is lacking. Knowing how many elephants there are and how many poachers kill is important, but on their own, such data lack context. We collated savanna elephant count data from 73 protected areas across the continent estimated to hold ~50% of Africa’s elephants and extracted densities from 18 broadly stable population time series. We modeled these densities using primary productivity, water availability, and an index of poaching as predictors. We then used the model to predict stable densities given current conditions and poaching for all 73 populations. Next, to generate ecological benchmarks, we predicted such densities for a scenario of zero poaching. Where historical data are available, they corroborate or exceed benchmarks. According to recent counts, collectively, the 73 savanna elephant populations are at 75% of the size predicted based on current conditions and poaching levels. However, populations are at <25% of ecological benchmarks given a scenario of zero poaching (~967,000)—a total deficit of ~730,000 elephants. Populations in 30% of the 73 protected areas were <5% of their benchmarks, and the median current density as a percentage of ecological benchmark across protected areas was just 13%. The ecological context provided by these benchmark values, in conjunction with ongoing census projects, allow efficient targeting of conservation efforts.

## Introduction

There are alarming statistics on extinction and the areas over which still extant species have lost their habitats [1, 2]. Savanna Africa is no exception. Human disturbance excludes many species from large fractions of their historical range [3, 4], and poaching threatens species, even where they are supposedly protected [5, 6]. Some of the protected areas that cover 13% of Africa struggle to stave off large mammal declines [7]. Such statistics on extinction and habitat loss are necessary, but not sufficient. They do not capture how much rarer some species are now, even within protected areas that aspire to maintain natural habitats.

Unsustainable poaching claimed 6.8% of Africa’s elephants (*Loxodonta africana*) annually from 2010–2012, upwards of 100,000 elephants [6]. These losses matter because elephants have an outsized role in savanna ecosystems. They affect the structure of the physical environment, disperse seeds, create microhabitats, and tend to dominate mammalian biomass [8–10]. Elephant presence engenders greater species diversity [8, 9]. Thus, human-mediated reductions in elephant densities will have cascading influences on other savanna species.

Unsurprisingly, there are frequent elephant counts, and elephants have the most comprehensive database on the conservation status of any mammal species [11, 12]. Despite this, there are no estimates of how many elephants we should expect there to be. How big would particular populations grow if afforded the chance? The lack of context is a serious omission given the ecosystem importance of elephants and the consequent imperative to conserve elephants effectively. The aim of this paper is to provide such estimates—ecological benchmarks against which to evaluate population counts and trends.

Elephant numbers are now certainly lower than what they should be. Here we ask: by how much? We model stable densities extracted from time series, relating them to primary productivity, water availability, and an index of poaching pressure. For savanna elephant populations in protected areas across Africa, we then predict stable densities based on both current conditions and a scenario of minimal poaching to generate ecologically relevant benchmarks. Finally, we compare recent population estimates against these ecological benchmarks to assess the extent to which elephant numbers are depleted.

## Results

We compiled counts for savanna elephants from IUCN category I-VI protected areas larger than 1,000 km^2^. This yielded 73 populations covering a total 529,363 km^2^ across 21 countries (see Supporting Information Materials and Methods and Figure A in S1 File). Elephants in these protected areas represent half of the continent’s estimated 473,386 elephants, the remainder attributable to smaller protected areas, unprotected regions, and forest (*Loxodonta cyclotis*) or hybrid elephants this study does not consider [12].

Under natural conditions, elephant population size should move towards and fluctuate around an environmentally-mediated equilibrium level [13–15]. To identify such equilibria, we used a model selection framework to characterize 23 time series by the AIC_c_-selected best of five candidate growth models [14, 16] (Figure B, Tables A and B in S1 File). Classically, we expected asymptotic growth patterns, particularly in populations recovering after culling or poaching, but we expected time series to exhibit different patterns depending on the time scale of data relative to the population’s history. Ongoing influences such as poaching or habitat destruction could result in populations in decline. Thus, candidate models included 1) logistic and 2) Gompertz growth (asymptotic models where the population grows until it reaches stability, e.g. after recovery from high poaching or culling); 3) the null model (where a lack of trend is assumed to represent stability); and 4) linear or 5) exponential change (where an asymptote is not apparent, e.g. a population in decline or an early stage of recovery). Neither logistic nor Gompertz growth were selected best for any population, while exponential change was selected for four populations and linear change for one. We extracted stable densities and their standard errors from the 18 populations where the null model was selected to be the best (Figure B, Tables A and B in S1 File). Model selection for the null model was robust under Monte Carlo simulation of time series incorporating uncertainty in counts (Table B in S1 File), and none of these 18 populations exhibited significant linear change over time.

**Fig 1:**
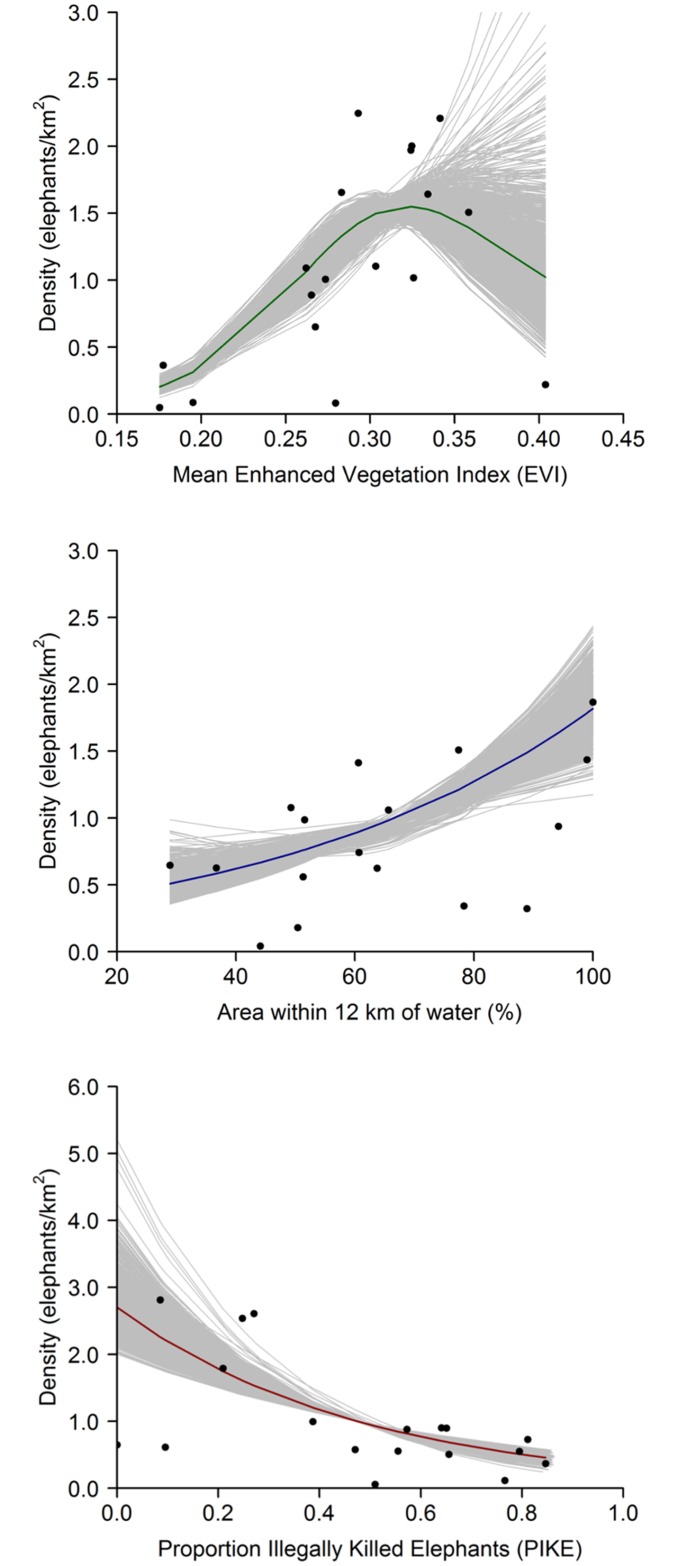
A GAM explains extracted stable densities from time series of 18 savanna elephant populations. The component smooth functions of mean EVI, percentage of protected area within 12km of water, and PIKE of the selected best GAM are represented by solid lines transformed to the response scale of density (elephants/km^2^). Points represent partial residuals of the 18 extracted densities, and grey lines represent 1,000 iterations to account for uncertainty.

We used a generalized additive modeling (GAM) framework to explain these stable densities. A GAM including primary productivity (mean enhanced vegetation index, EVI), water availability (percentage of protected area within 12km of water), and an index of poaching (proportion of illegally killed elephants, PIKE) explained 84% of variation in stable densities and performed well under cross-validation (Fig 1, Table E in S1 File). Primary productivity and water availability are known determinants of elephant distribution, fecundity, and survival [13, 14, 17–21]. They are ecological factors that should contribute to maintaining broad population stability. The inclusion of an index of poaching in our model shows that ecological factors alone did not determine stable densities in our study.

No data points had high leverage, although one, Murchison Falls National Park, was found to be influential (Cook’s distance >1). Murchison Falls had the highest EVI in the training data (0.404) but a relatively low stable density (0.182 ± 0.048 elephants/km^2^). We tested the impact of excluding this data point in modeling and found that predictions of stable density for populations within the range of EVI of the new training data were correlated with those from the original model (Figure D in S1 File), and the general conclusions of our analyses remained the same. However, excluding the point resulted in a continual increase of stable density with EVI (Figure D in S1 File), a pattern that is likely unrealistic [22, 23], and generates unreasonably high density estimates for high EVI populations (Figure D in S1 File). Contrastingly, including Murchison Falls pulls down density estimates for high EVI populations and yields appropriately wide prediction intervals indicating large uncertainty for high EVI populations (Fig 1). We have no reason to doubt the veracity of data for Murchison Falls, so we present results including it. However, we note that additional high EVI points would be valuable in refining the model.

We expected our model to provide realistic predictions of the approximate stable densities that savanna elephant populations should reach given current poaching levels. Of course, populations may not yet have reached stability after past influences, such as die-offs [24, 25] or culling [26], but the most recent population estimates for the 73 protected areas in the database [12] were correlated with predicted stable densities (r_s_ = 0.60, p<0.001). Indeed, recent estimates suggest a collective 237,515 elephants in the 73 protected areas assessed, a reassuring 75% of the modeled value of 317,314 [12] (Table F in S1 File).

**Fig 2:**
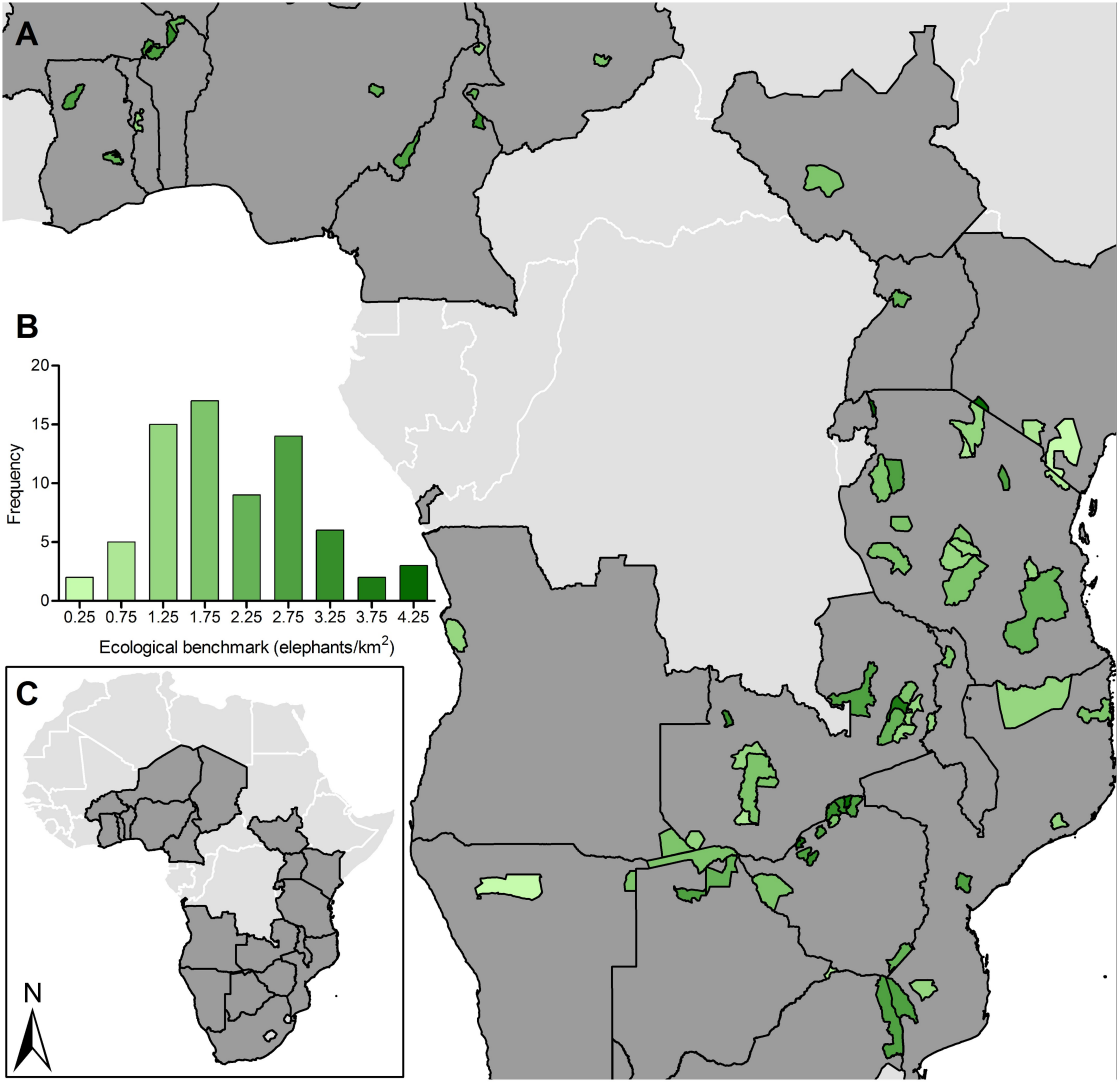
Predicted ecological benchmark density for savanna elephant populations across 73 protected areas differed according to EVI and water availability given minimized poaching. **(A)** Green fill color indicates predicted ecological benchmark density as shown in **(B)** the frequency distribution where densities on the x-axis represent bin centers. **(C)** Twenty-one countries are covered by protected areas in this assessment. See Figure E and Table F in S1 File for the 95% prediction intervals of predicted ecological benchmarks.

We used the model to predict stable densities for the same populations under a scenario of minimal poaching. For each of the 73 populations, we set PIKE = 0, equivalent to the minimum observed in the original training data (i.e. 0.00 in Etosha National Park), to predict ecological benchmarks. By this, we mean the population densities or sizes we expect in each protected area if ecological factors, rather than poaching, determined population dynamics. Under a scenario of current ecological conditions but minimum poaching, ecological benchmark densities ranged from 0.23–4.30 elephants/km^2^ with a median 1.93 elephants/km^2^ (Fig 2, Figure E in S1 File).

**Fig 3:**
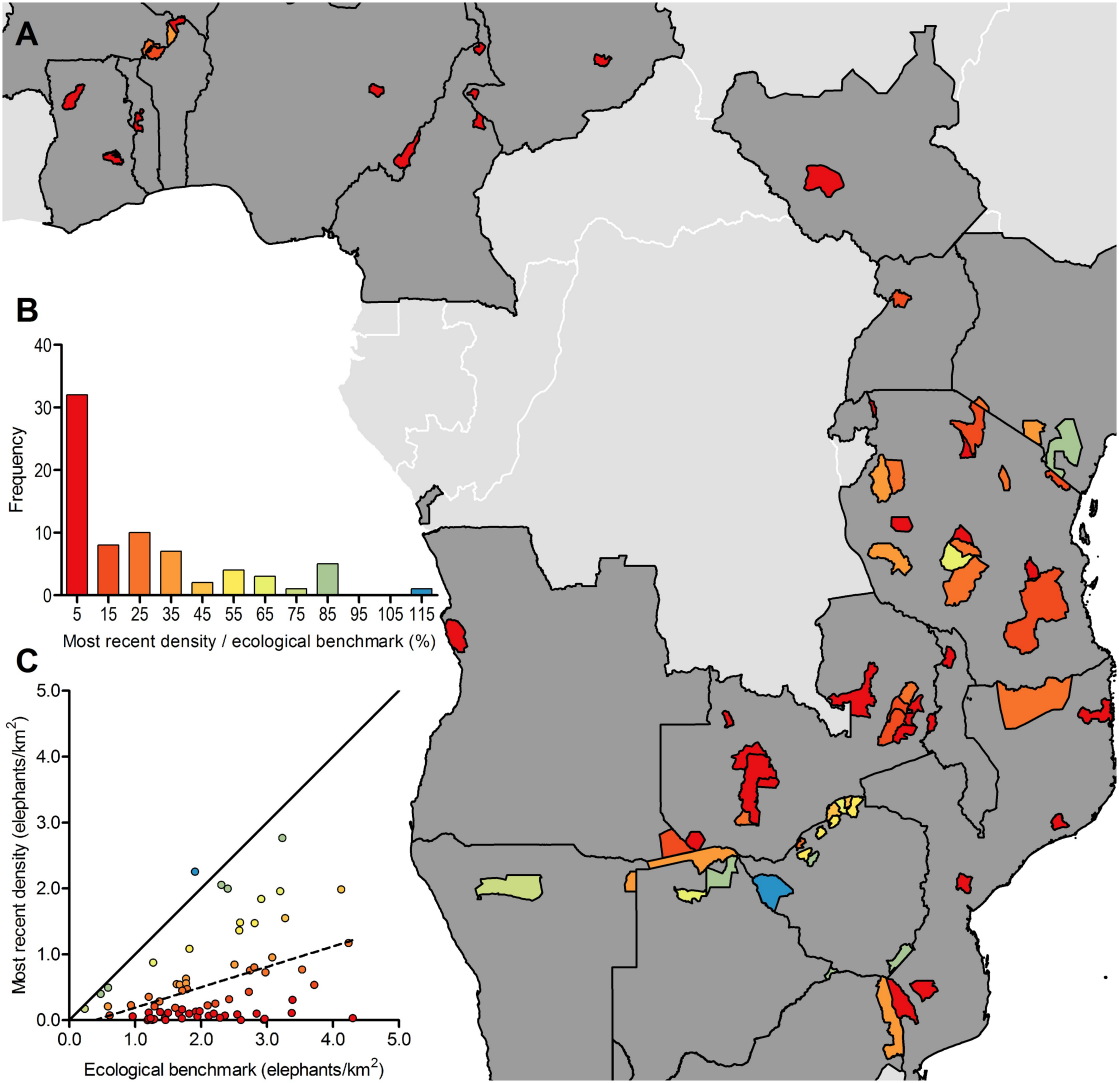
Most recent density estimates as a percentage of predicted ecological benchmark densities for savanna elephant populations across 73 protected areas indicate most populations are far from ecological benchmarks. **(A)** Fill color illustrates most recent density estimates as a percentage of predicted ecological benchmark density as indicated in **(B)** the frequency distribution where percentages on the x-axis represent bin centers. **(C)** Populations plotted according to the same color scheme show that while few populations are near their predicted ecological benchmark (indicated by the solid 1:1 line), recent density estimates increased with predicted ecological benchmark density (dashed line: y = 0.31x-0.12, F_1,71_ = 16.38, p<0.001, R^2^ = 0.19).

We assessed the most recent density estimate for each population as a percentage of its predicted ecological benchmark given minimal poaching. Only six protected areas had populations at ≥75% of their respective ecological benchmarks (Chirisa, Gonarezhou, and Hwange in Zimbabwe; Chobe and Tuli in Botswana; and Tsavo in Kenya, Fig 3, Table F in S1 File). Although protected areas with higher ecological benchmarks tended to have higher recent density estimates (Fig 3), across the 73 protected areas assessed, the median recent density estimate as a percentage of ecological benchmark was just 13.35%. The lowest value was 0.07%. In 32 of 73 protected areas, population estimates were ≤10% of their predicted ecological benchmark; in 22 protected areas, they were ≤5% of benchmarks.

**Fig 4:**
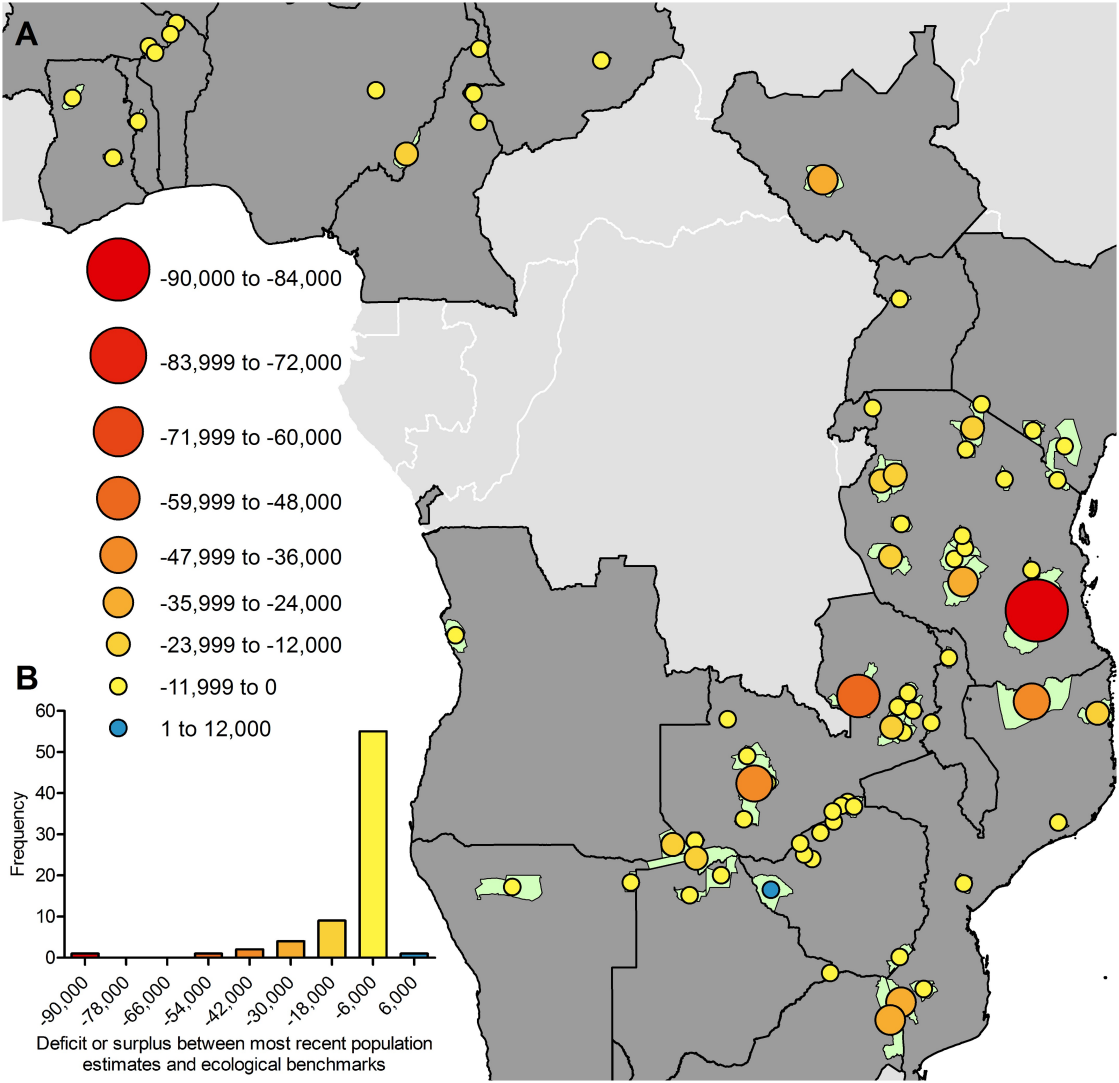
Of 73 protected areas assessed, 72 had a deficit of elephants compared to ecological benchmarks. (A) Subtracting predicted ecological benchmark population size from corresponding recent population estimates yields deficit or surplus estimates for each protected area, illustrated by size and color of circles (net deficit = 729,505). (B) A frequency distribution, where x-axis represents bin centers, illustrates that few populations have large deficits, while most are missing fewer than 12,000 elephants. See Table F in S1 File for the 95% prediction intervals of population deficits/surpluses.

We converted densities to population sizes and subtracted the predicted ecological benchmark for each protected area from the most recent population estimate (Fig 4, Table F in S1 File). Of 73 populations, 72 had an elephant deficit. However, protected areas in the analysis range in size (1,020–47,666 km^2^), so those with the highest discrepancy between recent and ecological benchmark density (Fig 3) do not necessarily have the highest deficit of elephants (Fig 4) and vice versa.

Therefore, while the median recent density as a percentage of ecological benchmark was 13.35% across the 73 protected areas assessed, in terms of cumulative numbers, recent population estimates (237,515) total just 24.56% of the cumulative ecological benchmark (967,014, (Figure F in S1 File)). This is a deficit of ~730,000 elephants. Comparing the ecological benchmark value under minimal poaching to the previously discussed predicted asymptotic total given current poaching levels (317,314), it is clear that poaching is responsible for the majority of the deficit.

## Discussion

Elephants have major impacts on savanna ecosystems. Consequently, human-mediated reductions in elephant densities will have cascading influences on many other species and, likely, on ecosystem processes. In protected areas that seek to minimize human influence and allow natural ecological processes to occur, elephant populations should tend towards densities where numbers will be relatively stable. Such numbers might serve as benchmarks for conservation managers. Despite resources dedicated to counting elephants, an ecological benchmark by which to evaluate counts was lacking. The models presented here not only predict the size at which elephant populations should stabilize given current environmental conditions (EVI and water availability) and PIKE, but also allow exploration of future scenarios such as climate change, water management, or changes in poaching. We explored the latter—by setting PIKE to zero, we predicted ecological benchmark densities for 73 protected areas and compared these benchmarks to recent counts.

Recent population estimates from the 73 protected areas we assessed collectively totaled <25% of the predicted ecological benchmark. We caution that the situation would likely appear much worse had it been possible to assess elephants across their entire known range, to include forest elephant populations, or to get more recent estimates that reflect the recent uptick in poaching. (The median year of the most recent estimates was 2011, with several populations last surveyed before the uptick in poaching since 2007 [27] (Table F in S1 File)).

Overall, 70% of the current distributional range of African elephants may fall outside of protected areas [11]. Yet, protected areas should be the stronghold for elephants on the continent, given that they should receive a concentration of funding, management, and law-enforcement. Accordingly, while the 73 protected areas in this analysis represent just 28% of the total range over which forest and savanna elephant populations have been assessed by the African Elephant Database (AED), their savanna elephant populations represent 50% of the total estimate of forest and savanna elephants combined [11, 12]. That populations are so far from ecological benchmarks, even within protected areas, is concerning. It bodes poorly for elephants and their ecological roles, within and beyond protected areas.

While some of the ecological benchmark predictions may seem high, the shifting baseline effect—a generational amnesia due to lack of experience about past conditions—likely shapes perceptions [28]. Although estimates of elephant population sizes before the effects of pervasive ivory poaching are limited, many of those available correspond to or exceed our median predictions of ecological benchmarks (see Table F in S1 File for associated prediction intervals). For example, Selous Game Reserve had an estimated 100,000 elephants in 1976 [29], while we predicted an ecological benchmark of 100,103. In 1972, Mkomazi National Park had an estimated 2,067 elephants [30]; we predicted 2,118. A total count in 1966 in Murchison Falls recorded 9,400 elephants [31]; our estimated benchmark was 9,308. In the Luangwa Valley and Tsavo East and West National Parks, elephant population increases in the 1960s and early 1970s were partially attributed to compression from surrounding landscapes following human population growth [32, 33]. Conceivably, immigration from surrounding areas could have resulted in population sizes larger than what would have developed otherwise. In 1973, South Luangwa had an estimated 31,600 elephants, and North Luangwa had 17,700 [33]. We predicted ecological benchmarks of 19,893 and 8,114 respectively. Six estimates of elephant population size in Tsavo East and West National Parks between 1962 and 1973 averaged 15,751 elephants [30], while we predicted an ecological benchmark of 9,568.

The predicted stable densities presented here provide a benchmark for assessing conservation and management success. Models are mere representations of reality and must assess the inevitable and surely considerable uncertainties. This is reflected in the relatively wide, asymmetrical 95% prediction intervals calculated via Monte Carlo simulation to include and combine uncertainty in counts, stable density estimates from time series, and modeled PIKE estimates, as well as model uncertainty in the GAM to predict stable densities. At any given site, the relatively simple model presented here, based on just three variables, may be an incomplete representation. Furthermore, predicted stable densities are based on current conditions of primary productivity and water availability. These may have been different in the past and may change in the future. Additionally, even without poaching, ecological benchmarks encompass elements of human interference that should be considered in developing conservation goals. Most protected areas in our analysis are large and unfenced. Surrounding human settlements and land-use may restrict elephants’ seasonal movements and access to resources. Additionally, some protected areas encompass human settlements or land use. Moreover, many protected areas contain artificial waterholes that may increase stable density over what one would otherwise predict for that area.

Nonetheless, our result that two of the three largest deficits between ecological benchmarks and recent estimates come from Tanzania’s Selous and Mozambique’s Niassa Game Reserves (with respective deficits of ~89,000 and ~46,000 elephants, Fig 4) agrees with DNA analysis [34]. This analysis traced 86–93% of savanna elephant ivory confiscated between 2006 and 2014 to a geographic source in southeastern Tanzania and northern Mozambique [34]. The deficit of ~50,000 elephants in the Bangweulu System, a patchwork of national parks and human-inhabited game management areas, supports the suggestion that the remnant population of just 136 elephants is in need of conservation intervention [35].

We provide an ecological context to population counts conducted across the continent, including the pan-African census [36]. The problem of poaching is widely known and discussed as a serious threat to the species [6, 27], and by extension, savanna ecosystems [37, 38]. Our results quantify the problem against an ecologically relevant benchmark rather than against simplistic population trends or the zero-point of extinction. This allows for targeted, goal-driven conservation measures. For example, considering the 72 protected areas with fewer elephants than they should have, the cumulative deficit of the 10 highest-deficit protected areas (~373,000) exceeds that of the other 62 protected areas combined (~356,000). Conservation efforts targeted at high-deficit populations would more efficiently boost total elephant numbers in the long run. Conversely, conservationists concerned with restoring the role of elephants in savanna ecosystems might focus on protected areas where current populations represent the lowest percentages of predicted ecological benchmarks, even though relatively small protected areas contribute little to elephant numbers on a continental scale.

## Methods

### Ethics Statement

All aspects of the study were completed in accordance with international and national guidelines and the Animal Ethics Committee of University of Pretoria (AUCC-040611-013).

#### Extracting stable densities from time series

We compiled count data on savanna elephants from IUCN category I-VI protected areas ≥ 1,000 km^2^ (see Supporting Information Materials and Methods and Figure A in S1 File). From these 73 populations, 23 had adequate time-series for assessing population trends. That is, they had populations of ≥ 500 individuals with ≥ 5 counts with high survey reliability (A or B [11]) since 1989.

We followed published methods [14, 16] and a model-selection approach to assess the dynamics of the 23 populations for which we had an adequate time-series. Based on different types of population changes that could be expected (as discussed in the Results), we fitted five alternative candidate models to determine the most likely trend in density over time for each population: null (no trend), linear change, exponential change, and two asymptotic increases experiencing logistic or Gompertz dynamics (see Table A in S1 File for equations). To fit models, we used “minpack.lm” package in the R language [39] which uses least squares estimation. We calculated Akaike Information Criteria adjusted for small sample size (AIC_c_) to indicate the most likely model. Where a linear or exponential model was selected over a null (no trend), logistic, or Gompertz model, populations were excluded from further analysis. Where the null model was selected, we confirmed that no populations had a significant change over time (p>0.05 for linear trend). While some population estimates came from total counts, many came from sample counts with associated error estimates. To incorporate uncertainty associated with counts, both in model selection and final estimates of stable densities, we used Monte Carlo methods to simulate 1,000 time-series for each population based on draws from the normal distribution given each count’s estimate and standard error (SE). (Note, SE = 0 for estimates from total counts). We assessed how robust model selection was given count uncertainty as the proportion of the 1,000 runs in which the selected best model matched the selected best model for the original data. For each population, we calculated stable density and its SE from the results of fitting the selected best model to each of the 1,000 simulated datasets.,

#### Predicting stable densities for savanna elephant populations

We used a negative binomial generalized additive modeling (GAM) approach to calculate an explanatory model for extracted stable population sizes allowing for nonlinear response shapes [40]. GAMs were fitted using the “mgcv” package from the R language [39]. The negative binomial, rather than Poisson, was used due to over-dispersion (dispersion parameter for the negative binomial distribution = 3.10) [41]. To avoid overfitting, we used a thin plate regression spline smoothing function with an upper limit of three smoothing knots per explanatory variable [41]. We used the generalized cross-validation method to fit smoothers. Explanatory variables were mean EVI (2000–2013) (*EVI*), proportion of area within 12km of water (*prop_12_water*), and PIKE (*PIKE*) (from empirical data [42] or modelled where no PIKE data was available [6, 43] (Tables C and D and Figure C in S1 File) for each protected area (see Supporting Information Materials and Methods in S1 File for details of each variable and model specifications). The natural log of the area of each site (km^2^) was included as an offset variable. We constructed three candidate models, *EVI* + *prop_12_water*, *EVI* + *prop_12_*water + *PIKE*, and a null model, and we excluded models with other combinations of these variables as ecologically irrelevant. We calculated AIC_c_, ΔAIC_c_, AIC_c_(wi), R^2^, adjusted R^2^ (R^2^_adj_), and the correlation coefficient (COR) for each candidate model. Due to a relatively small sample size, we used leave-one-out cross-validation (LOOCV) to calculate the cross-validation correlation coefficient (cvCOR) and Wilmott’s index of agreement (D) to assess the predictive performance of each model. We calculated the mean bias error (MBE) to measure potential skew in the distribution of errors. We selected the model with the optimal set of explanatory variables based on lowest AIC_c_. For each data point, we calculated hat values, a measure of leverage where hat > 3 times the mean hat value indicates high leverage, and Cook’s distance, a measure of influence where values > 1 warrant further inspection [41].

We used the selected GAM to predict stable population sizes for the 73 protected areas in the analysis based on current conditions of EVI, distance to water, and PIKE and on a scenario of minimum poaching where PIKE = 0, the lowest in the dataset. The latter represents the ecological benchmark for each protected area. We then compared recent estimates for each population to its predicted stable density given current conditions and ecological benchmark.

To generate realistic prediction intervals, we used Monte Carlo simulation to incorporate uncertainty in estimates of both stable densities extracted from time series and modeled PIKE values with model uncertainty from fitting the GAM. Based on the stable density estimate and its standard error for each of the 18 time series, we simulated 1,000 datasets from a normal distribution. Correspondingly, we simulated 1,000 datasets of modeled PIKE values given coefficient estimates and SE on the scale of the linear predictor of the generalized linear model to predict PIKE (Table D in S1 File). We then refitted the GAM to predict stable population size for each of these 1,000 datasets of simulated extracted stable densities and PIKE values. From each of the 1000 fits of the GAM, we incorporated model uncertainty by simulating from the posterior distribution of model coefficients of the GAM to generate 1,000 predicted stable population size values for each of the 73 protected areas in the analysis, both given current conditions and a scenario of minimal poaching. For each simulated dataset, we also calculated the difference between a population’s predicted stable size and its most recent estimate, as well as an overall total and deficit for all 73 populations. Thus, median values, standard errors and asymmetrical 95% prediction intervals (i.e. the spread of 95% of estimated values, excluding the top and bottom 2.5%) were calculated based on 1x10^6 estimate predictions for each protected area—i.e. 1000 simulated prediction datasets incorporating model uncertainty from each of the 1000 GAMs refit to the simulated datasets incorporating uncertainty in stable densities and PIKE. As expected, median estimates were approximately equal to the values predicted from the original GAM, but the 95% prediction intervals better represent the true uncertainty around the estimates. Throughout the main text, estimates of stable densities based on current conditions and PIKE, ecological benchmark densities where PIKE = 0, population deficits or surpluses, and collective totals of these across the 73 protected areas assessed refer to median values from the 1x10^6 runs based on Monte Carlo simulations to incorporate uncertainty. Full simulation results, SE’s, and 95% prediction intervals are available in the supporting information.

Not all uncertainty could be incorporated. Empirical PIKE values are subject to error and potential bias beyond the scope of this study, but they provide the best available index of poaching intensity [6]. We minimized stochastic variability in PIKE by amalgamating carcass counts among all the years of data collection at each site (up to 12 years, 2002–2014) and excluding PIKE estimates from sites where the amalgamated carcass count was <20. Additionally, although we used the best count data available and incorporated uncertainty for all sample counts, more count data points would likely reduce prediction intervals and make model selection more robust. In the future, as more counts accumulate for populations, additional populations can be assessed for stability, and stability estimates from populations already in our dataset can be refined.

## Supporting information

S1 File Additional methods and supporting results and references. This file contains Supporting Information Materials and Methods; Figure A (study schematic), Figure B (23 time series and best-fit population models); Figure C (partial residual plots of components of model averaged GLM to explain PIKE values from MIKE sites); Figure D (comparison of GAMs to explain extracted stable densities with and without influential points); Figure E (histogram of ecological benchmark estimates for each of 73 protected areas illustrating results of Monte Carlo simulation to incorporate uncertainty); Figure F (histogram of cumulative ecological benchmark across 73 protected areas recalculated for each of 1x10^6 runs from the Monte Carlo simulation to incorporate uncertainty); Table A (alternative candidate models to describe the population dynamics of 23 time-series populations); Table B (summary information on time series and extracted stable density and SE); Table C (summary information for 43 MIKE sites and the explanatory variables used to explain PIKE); Table D (Summary statistics and variables included in most likely quasi-binomial generalized linear models to explain PIKE for 43 MIKE sites across Africa and final predictive average model used to generate PIKE estimates for non-MIKE sites); Table E (selection parameters of candidate generalized additive models explaining variation in extracted stable population size for 18 populations); Table F (summary information, predicted stable density (give current PIKE), and ecological benchmark density (given zero PIKE), and comparisons between most recent density and population size estimates and ecological benchmark density and population size for 73 protected areas across 21 countries); and Supporting Information References.
(DOCX)
