# Supplementary material for: Savanna elephant numbers are only a quarter of their expected values
Source: PLoS One. 2017 Apr 17;12(4):e0175942. doi: 10.1371/journal.pone.0175942 (PMC5393891; doi:10.1371/journal.pone.0175942)
Supplement: S1 File — This file contains Supporting Information Materials and Methods; Figure A (study schematic), Figure B (23 time series and best-fit population models); Figure C (partial residual plots of components of model averaged GLM to explain PIKE values from MIKE sites); Figure D (comparison of GAMs to explain extracted stable densities with and without influential points); Figure E (histogram of ecological benchmark estimates for each of 73 protected areas illustrating results of Monte Carlo simulation to incorporate uncertainty); Figure F (histogram of cumulative ecological benchmark across 73 protected areas recalculated for each of 1x10^6 runs from the Monte Carlo simulation to incorporate uncertainty); Table A (alternative candidate models to describe the population dynamics of 23 time-series populations); Table B (summary information on time series and extracted stable density and SE); Table C (summary information for 43 MIKE sites and the explanatory variables used to explain PIKE); Table D (Summary statistics and variables included in most likely quasi-binomial generalized linear models to explain PIKE for 43 MIKE sites across Africa and final predictive average model used to generate PIKE estimates for non-MIKE sites); Table E (selection parameters of candidate generalized additive models explaining variation in extracted stable population size for 18 populations); Table F (summary information, predicted stable density (give current PIKE), and ecological benchmark density (given zero PIKE), and comparisons between most recent density and population size estimates and ecological benchmark density and population size for 73 protected areas across 21 countries); and Supporting Information References. (DOCX) [file pone.0175942.s001.docx]

Supporting Information: S1 File

Savanna elephant numbers are only a quarter of their expected values

Ashley S. Robson, Morgan J. Trimble, Andrew Purdon, Kim D. Young-Overton, Stuart L. Pimm, Rudi J. van Aarde

correspondence to: rjvaarde@zoology.up.ac.za

**This file includes:**

Supporting Information Materials and Methods

Figures A, B, C, D, E, F

Tables A, B, C, D, E, F

Supporting Information References

Supporting Information Materials and Methods

Compiling the population datasets

Figure A summarizes our procedures. Data on savanna elephants came from the African Elephant Database [12], our own databases (see [44] for details), and internet searches. We excluded suspected forest or hybrid elephants [11, 12, 45]. To generate a dataset we called *time-series populations*, we applied a rigorous set of selection criteria:

1. We standardized protection status by only including populations located within protected areas of IUCN categories I to VI.
2. We included populations only when the survey area (via [12]) matched the spatial boundary of the associated protected area (via World Protected Areas; www.protectedplanet.net).
3. To ensure that we captured population processes at the appropriate spatial and numerical scale, we limited this dataset to populations numbering >500 individuals from protected areas >1,000km^2^ (e.g. [46]).
4. While, AED compiles population estimates generated by various survey methods [12], we minimized error within the dataset by only including estimates with a survey reliability of A or B [11].
5. Finally, we only included populations in this dataset for which there were five or more population estimates between 1989, the year the international ban on the trade in ivory came into effect, and 2013. We excluded periods when elephants were culled due to artificial controls on population growth, but included periods following cessation of culling when populations would be recovering and expected to follow a theoretical growth model mediated by resource availability [13-15].

These selection procedures yielded 23 time-series populations. We further generated a second dataset we call *known AED populations* from populations that did not meet all the criteria (1) to (5). We retained criteria (1), (2), and the protected area had to be >1,000km^2^. However, we relaxed the 500-individual criterion to any protected area known to have elephants, regardless of its population size. Likewise, we relaxed the survey reliability criterion and used data regardless of the method used to derive them. Finally, we incorporated time-series populations for which we could not extract a stable density (see next section) into the known AED populations dataset. This selection procedure returned 55 known AED populations, including time-series populations without an extracted stable density (Table B). Combined, we produce a dataset of 73 savanna elephant populations from 21 countries across central, east, southern, and west Africa.

Variables to explain variation in stable densities

In the GAM modeling framework to explain stable densities, we used two variables to capture the primary resources of food and water and a third—an index of poaching—to quantify the human threat to populations within protected areas. The basis for inclusion and methods for calculation are as follows:

*Primary production*

The amount of plant growth—primary production—affects elephant habitat selection [47], distribution [19], fecundity [17], and survival [18, 20]. It seems likely it will also determine stable densities. We used the Enhanced Vegetation Index (EVI) as an index of productivity [48], which has, along with Normalized Difference Vegetation Index (NDVI), been used widely in ecological studies and for elephants specifically [17, 18, 47, 49, 50, 54]*.* We chose EVI over NDVI because it overcomes many of the contamination problems present in NDVI and does not become saturated as easily in high-biomass areas [48, 51]. We downloaded monthly EVI layers (250m resolution) from <http://reverb.echo.nasa.gov/> and calculated a long-term mean EVI (2000-2013) for each protected area. The long-term mean was most appropriate to capture broad scale differences among protected areas over long stretches of time across a continental scale. We excluded all water pixels from the analyses and set all EVI values <0.05 (indicative of non-vegetated areas) to 0.05 [52]. Preliminarily, we considered using mean annual rainfall from 1950 to 2000 (WorldClim v1.4) [53] or mean tree cover (2000-2013) (MOD44B downloaded from <http://reverb.echo.nasa.gov/>) as additional indices of vegetation productivity. We found that rainfall and tree cover were highly correlated with EVI across protected areas (r = 0.79 and r = 0.85 respectively) and therefore excluded them from subsequent analyses in preference of EVI.

*Water availability*

We include the proportion of an area with water available to elephants as a variable in our model. Water is an essential resource for elephants. Its distribution influences the way individual elephants move [54], it dictates how much of an area is available for foraging [55], and consequently, can determine stable densities by mediating density-dependent effects [13, 14]. Density-dependent population regulation likely occurs when elephants are forced to commute far distances between forage and water sources [13, 14, 20].

Two global water layer products exist [56, 57]. Neither were appropriate for this study because small water bodies important for elephants (e.g. waterholes in Hwange National Park) were not detected either due to the coarse resolution of the raw data [56] or strict detection criteria [57]. Consequently, we used Landsat 8 imagery and supervised classification to generate our own fine-scale (30m) water distribution estimates for each of 73 protected areas. Based on the water distribution and telemetry data recorded across southern Africa, we estimated the proportion of each protected area available to elephants.

Satellite data: We downloaded freely available Landsat 8 data (30m resolution) from http://earthexplorer.usgs.gov. We used Landsat 8 data instead of Landsat 7 ETM+, which suffers from missing data due to the Scan-Line-Detector malfunction of the Landsat 7 ETM+ sensor. Furthermore, the 12-bit sensor can produce images with an improved signal-to-noise ratio and capture more discrete levels of electromagnetic radiation. This increases pixel classification precision and distinguishability from other pixels [58].

A dry season estimate of water distribution was the most appropriate for our assessment, as it represents a water estimate during the critical, potentially limiting season for elephants. Additionally, we expected that by using dry season Landsat 8 images, we could avoid cloud cover in images and improve our ability to detect water. We used estimates of mean monthly rainfall from 1950-2000 (WorldClim) to determine the mean lowest rainfall month for each site. We further recognized that water distribution might vary across years. Therefore, we downloaded one Landsat 8 image from the driest month in two separate years for each tile overlapping a site. Even during the dry season, some images, especially in east Africa, contained some cloud. We used the image with the least cloud cover, and as close to our desired date, as possible. We downloaded 254 Landsat 8 images.

*Image pre-processing, calculating indices and accounting for terrain shadow*. We converted all bands to at-surface-reflectance using ENVI (Exelis Visual Information Solutions, Boulder, Colorado). For our analyses, we considered 7 of the 11 bands: coastal, blue, green, red, near infrared (NIR), shortwave infrared 1 (SWIR 1), and shortwave infrared 2 (SWIR 2). We calculated an additional five indices to optimize the spectral decision space for detecting water: the normalized difference water index (NDWI) [59], two modified normalized difference water indices (MNDWI_36_ and MNDWI_37_) [60], the normalized difference vegetation index (NDVI) [61], and the normalized burn ratio (NBR) [62].

The similarity between terrain shadows and water can be a source of major errors when detecting water using satellite imagery [63]. To avoid them, we derived topographic slope and hill-shade from the 30m-resolution ASTER Global Digital Elevation Model (GDEM) v2.0 dataset [64]. Slope was calculated as the maximum rate of elevation difference between each pixel and its neighbors [65]. Hill-shade, representative of the sunlight received at each pixel and therefore an estimate of terrain shadow, was calculated by simulating solar geometry (altitude and azimuth) at the time of each Landsat 8 image’s acquisition. We only included pixels where slope was >20 degrees and hill-shade was <150 [57, 64]). The final composite of each tile therefore included 14 variables: coastal, blue, green, red, NIR, SWIR 1, SWIR 2, NDWI, MNDWI36, MNDWI37, NDVI, NBRI, slope, and hill-shade.

*Training data and random forest*. We used one image (LC81680772014226LGN00) from the central and southern regions of Kruger National Park to generate training data. We chose this region because the distribution of water is well documented and the image included all appropriate classification classes: 1) water, 2) sand, 3) terrain shadow, 4) burnt area, 5) vegetation, 6) cloud, and 7) agriculture/healthy vegetation. To capture a large enough sample of each class, we also generated training data in areas beyond park boundaries.

We used a random forest model for supervised classification of Landsat imagery [66]. Random forest uses classification trees with minimal bias and high variance in a voting process [67]. The method is recognized as a powerful classifier because it is efficient at handling a large number of input variables and can distinguish and make use of the most powerful variables [67]. Two important parameters to set in a random forest model are the number of decision trees and the number of variables split at each node. Here, we set the number of trees to 1,000, and the number of variables split at each node as the square root of the total number of input variables [68]. We trained our training scene composite image within the random forest model based on information from each variable extracted from 5,000 random sample pixels per classification class. According to out-of-bag error (OOB), a generalized error estimate for the whole model based on the classification of unused training data (1/3 of total training data) [67], the model had low classification error (OOB = 0.12%) across all classification classes. Classification error for the water class was low (0.002%) due to very slight confusion between water and terrain shadow. We used this random forest model to classify each of the 254 composite tiles.

*Post-random forest processing.* We separately extracted the water and cloud classes from each classified tile and identified matching pairs of tiles from different years (e.g. LC81680672013191LGN00 and LC81680672014194LGN00). We used the two-year overlap of water classes to determine water pixels that were common for both years, therefore accounting for possible inter-year water variation. We considered that water present during the dry season in both years was “permanent water” and available to elephants during wet or dry years or seasons. We buffered one year’s water layer by two pixels (60m) to allow for slight spatial variation in point water sources or rivers, and extracted water pixels from the second year that overlapped the buffer. The two-year overlapping process, while mostly to account for inter-year variability in water distribution, also functioned to remove pixels that were falsely classified as water in one of the years but not the other. For instance, in some tiles, burnt areas covered by smoke from fires, were falsely classified as water. It was unlikely that the same area would burn the next year, and therefore, there was no overlap between false water pixels. Although very few tiles contained cloud, we recognized that cloud cover could produce false-negatives for water. Therefore, where pixels were classified as water in one year, but classified as cloud in the other (i.e. cloud could have covered the water), we classified the pixel as water. The preliminary water layer tiles contained water pixels from the two-year water overlap and the cloud-water overlap.

*Validating preliminary water layer tiles.* We performed the validation of preliminary water layer tiles manually. While near impossible for a global level assessment, a manual validation was appropriate given the relatively small number of tiles (n=127). Human validation outperforms statistical validation in this case because the human eye can extract more information from an image (e.g. texture, shape) than a validation procedure only using the values associated with input variables. In ArcGIS, we merged all preliminary water layer tiles and converted all water pixels into points. This allowed for a better visualization of water distribution. We examined each study site at a 1:40,000 scale. We validated our estimated water layer against four sources: Google Earth 7 [69], Global Inland Water dataset [57], and the two raw Landsat 8 images used to generate the estimate. We converted the raw Landsat images to a false color composite (R=NIR, G=SWIR 1, B=Red) to better visualize water. Using these four validation sources, we inserted points where the preliminary water layer had clearly missed a water pixel, and deleted false-positives. This procedure likely generated the most accurate fine-scale water distribution estimate available for the 73 protected areas.

*Determining the proportion of protected areas available to elephants.* While water is a known limiting factor for elephants, estimates of how far elephants move from water, and therefore how much of an area is potentially available to elephants, are limited [e.g. 54, 70, 71]. We used telemetry data from 91 individuals (76 cows, 15 bulls) to derive an estimate of how far elephants tend to move from water, and thus the proportion of protected areas available to them. Individuals were captured and collared with Africa Wildlife Tracking GPS collars (model SM 2000E; African Wildlife Tracking, Pretoria, South Africa) between December 2002 and July 2012. Collared elephants were geographically representative of a large portion of the distributional range of elephants in southern Africa, including 11 protected areas: Caprivi Region, Chobe National Park, Etosha National Park, Kafue National Park, Khaudom Game Reserve, Kruger National Park, Limpopo National Park, Lupande Game Management Area, Mana Pools National Park, North Luangwa National Park, and South Luangwa National Park. Collars were programmed to relocate individuals at varying intervals, ranging from 1 to 24 hours, with most individuals having varying interval settings during the collaring period. Telemetry locations for the southern African core dry season (June through September) [19] were combined across years and all individuals, yielding 250,484 locations. We calculated the Euclidean distance between each telemetry location and the nearest water source, as determined above. Some 95% of telemetry locations were within 12 km of a water source. The area available to elephants for foraging is related to the maximum distance they can move from water. While elephants may occasionally move further, the proportion of each protected area within 12km of water should be an ecologically meaningful index of water distribution. In unfenced protected areas, elephants may have access to water or forage beyond the protected area’s boundaries. It is impossible to calculate the actual area over which elephants range without extremely fine scale, georeferenced total counts carried out across different seasons or a large number of collared elephants in each protected area. Neither exist. However, as counts and density estimates are also bounded by protected area demarcations, the proportion of each protected area within 12km of water is a useful proxy.

*Poaching pressure*

Poaching substantially influences elephant population dynamics in some parts of Africa [6]. Estimates of stable densities are likely not only a result of ecological factors but also of poaching pressure. Percentage Illegally Killed Elephants (PIKE) is a widely used index of poaching [6]. Out of 18 stable populations however, only 9 were from MIKE (Monitoring the Illegal Killing of Elephants) sites with associated PIKE estimates [42].

*Predicting PIKE for non-MIKE sites.* We used PIKE data from 43 MIKE sites across Africa to build a model that would allow us to predict PIKE for sites where no estimates existed. For each site, we combined PIKE data over all reporting years (2002-2014) to calculate a single amalgamated PIKE value. as we were interested in a long-term average as a covariate. Following the published quasi-binomial generalized linear modelling framework [6, 43], we used PIKE as the response variable and excluded populations where the total number of recorded carcasses was <20 [6, 43]. We included both forest and savanna elephant MIKE sites to increase sample size. We used the published best-model results [43, 72) to a priori select candidate country- and site-level explanatory variables (Table C): infant mortality rate (SEDAC, http://sedac.ciesin.columbia.edu/data/collection/povmap), site area (log transformed), mean Control of Corruption (1996-2013) (World Bank, http://info.worldbank.org/governance.wgi/index.asp), human density (AfriPop Project 2010, www.afripop.org), mean percentage tree cover (2000-2013) (MOD44B), mean Government Effectiveness (1996-2013) (World Bank), and mean Human Development Index (1980-2013) (United Nations Development Programme, http://hdr.undp.org/en/statistics/) (For a detailed description and the sources of each variable see 43, 72). We included three additional site-level variables. We calculated mean *Plasmodium falciparum* incidence rate (2000-2013) [67, http://www.map.ox.ac.uk], a variable potentially related to poverty [73, 74]. We tested a categorical variable of sub-species (savanna, forest, or both) to ensure including forest MIKE sites did not bias predictions for savanna sites. We added a variable related to scientific interest in elephants per site as a readily quantifiable proxy of investment, interest, and physical presence onsite; studies show that scientific investment in conservation biology research is not based on threat but on biases of geography, research infrastructure and funding, and personal interest (e.g. [75, 76]). We used the advance search on the Zoological Record database 1989-2013 (Ovid Technologies 2015) and a keyword search term to return publications for each site that mentioned elephants (Boolean search terms: “variations of site name” (e.g. “Zakouma National Park” OR “Parc National de Zakouma”) AND “elephant”). We took the number of publications returned as an index of scientific interest in elephants. Control of Corruption and mean *Plasmodium falciparum* incidence rate were highly correlated with Government Effectiveness (r = 0.85) and infant mortality rate (r = 0.70) respectively. As Control of Corruption and mean *Plasmodium falciparum* incidence rate singularly explained a larger proportion of variation in PIKE than their counterparts did, we excluded Government Effectiveness and infant mortality rate from subsequent analysis. We calculated candidate models for every combination of explanatory variables and used QAIC_c_ to determine the most likely set of models (ΔQAIC_c_ < 2) [77*]*. We performed model averaging on the most likely models to generate a final predictive model [43] (Table D, Figure C). Cook’s distance for all data points was <1, indicating no data point was highly influential on parameter estimation. Three points had relatively high leverage with hat values greater than three times the average, meaning their independent variable values were extreme—these were Chobe, Samburu-Laikipia, and Tarangire-Manyara. Their low Cook’s distance values (0.15, 0.02, and 0.02 respectively) confirm they are not, however, influential on model fitting, and they were retained [41]. The predictive model robustly explained PIKE, with a mean R^2^ of 0.82 for the two averaged models (Table D). We used this model to predict PIKE for the 9 non-MIKE “stable populations” and 43 non-MIKE “known AED populations”.

*Notes on variable selection*

Many additional variables could have been selected for testing, but given the limited sample size of 18 stable populations and the danger of overfitting and spurious results when too many variables are used [77], we limited this analysis to only those variables with the strongest theoretical underpinning as described above. Each of these three variables comprises a long-term average because the goal was to characterize inter-site differences on a continental scale rather than differences over time. Note that the time periods over which variables are representative was constrained by data availability and differ slightly among variables, but cover the relevant population survey data as closely as possible. As more elephant population data accumulate over time, it would be interesting to also analyze the impact of extreme values rather than averages, e.g. minimum EVI or a short-term spike in PIKE.


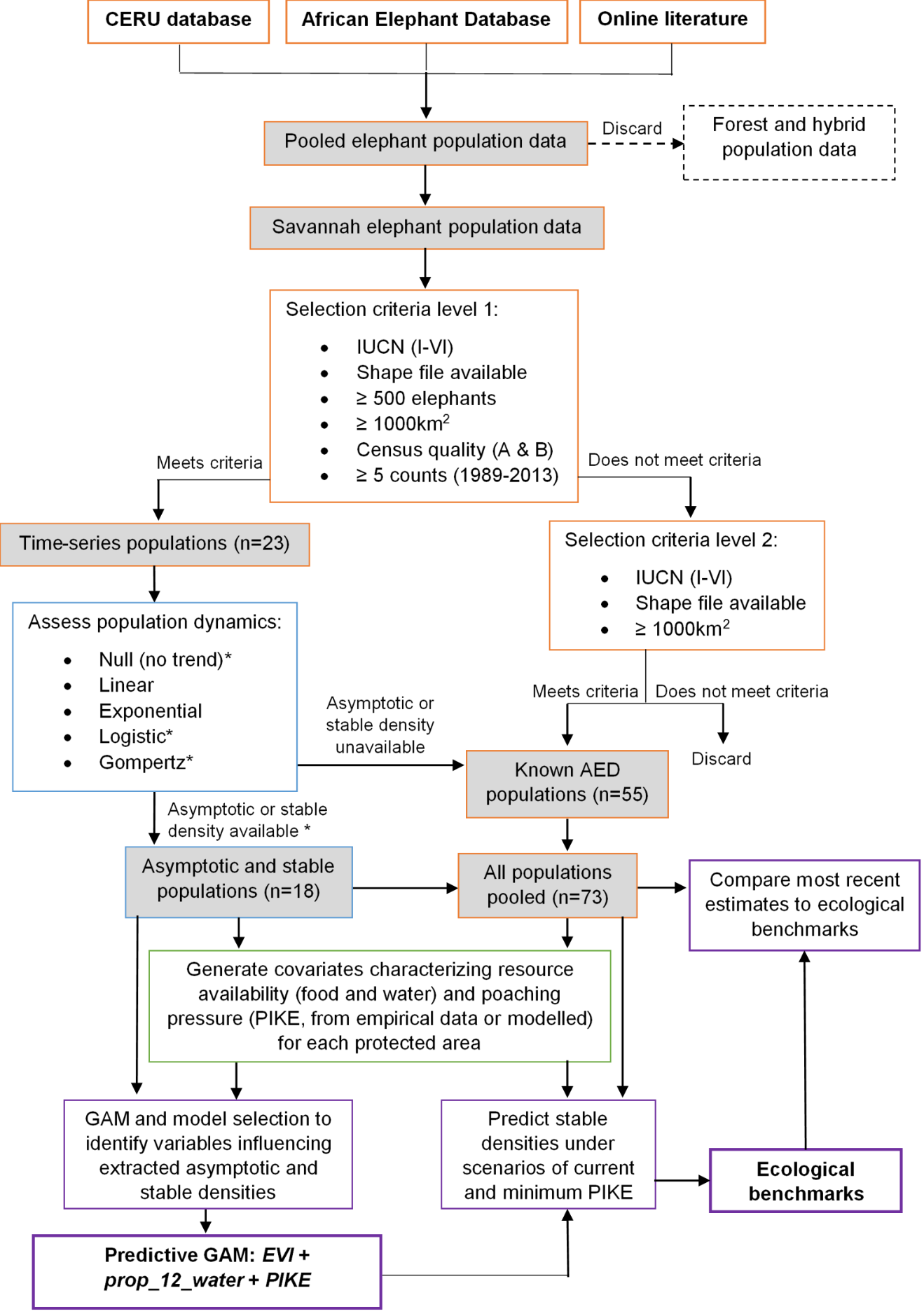


Figure A. Study Schematic. Illustrates process from data collection to final analysis. Orange outlined boxes correspond with the “Compiling Population Datasets” section of the supporting information, Blue outlined boxes are further explained in the section “Extracting stable densities from time series” in the main text. Green outlines boxes correspond to “Variables to explain variation in stable densities” in the supporting information. Purple outlined boxes correspond with the “Predicting stable densities for savanna elephant populations” in the main text.


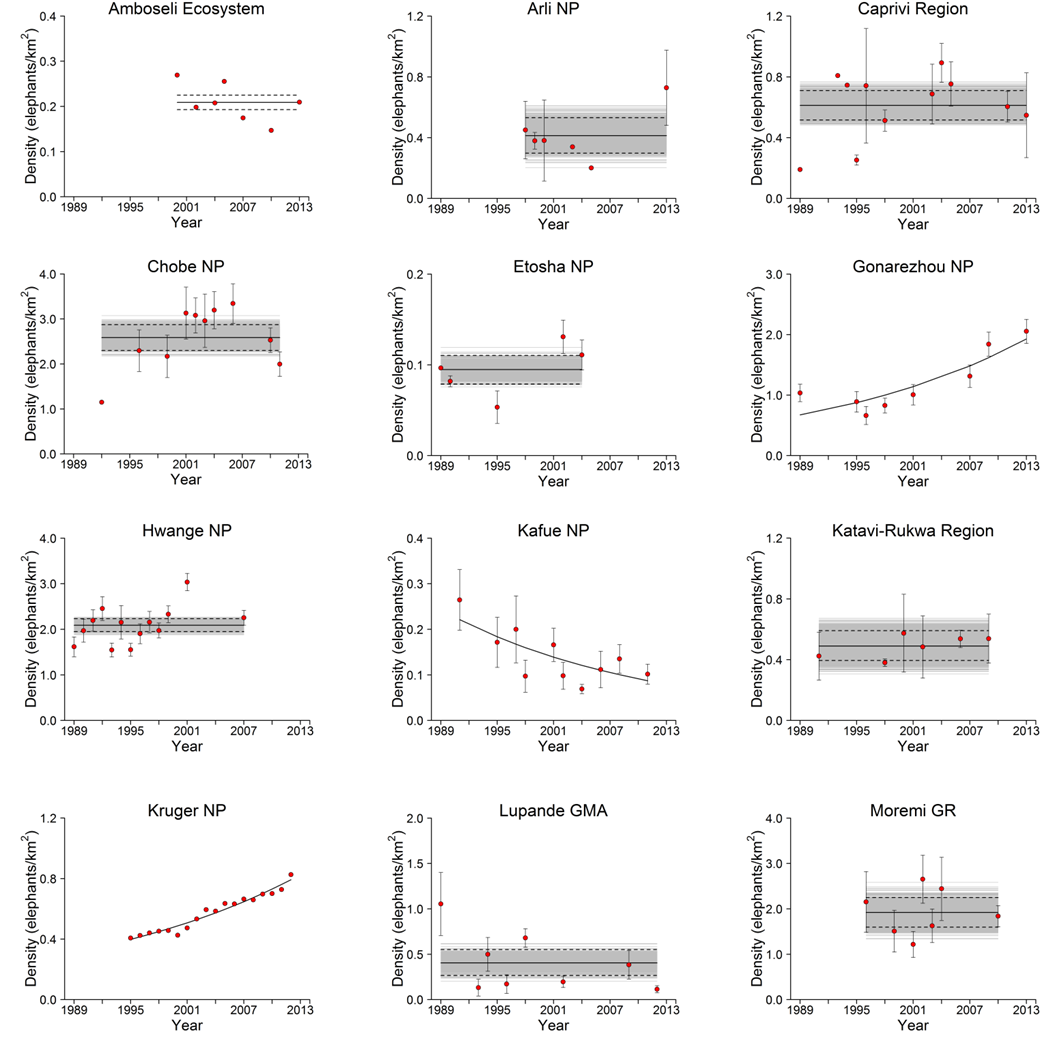


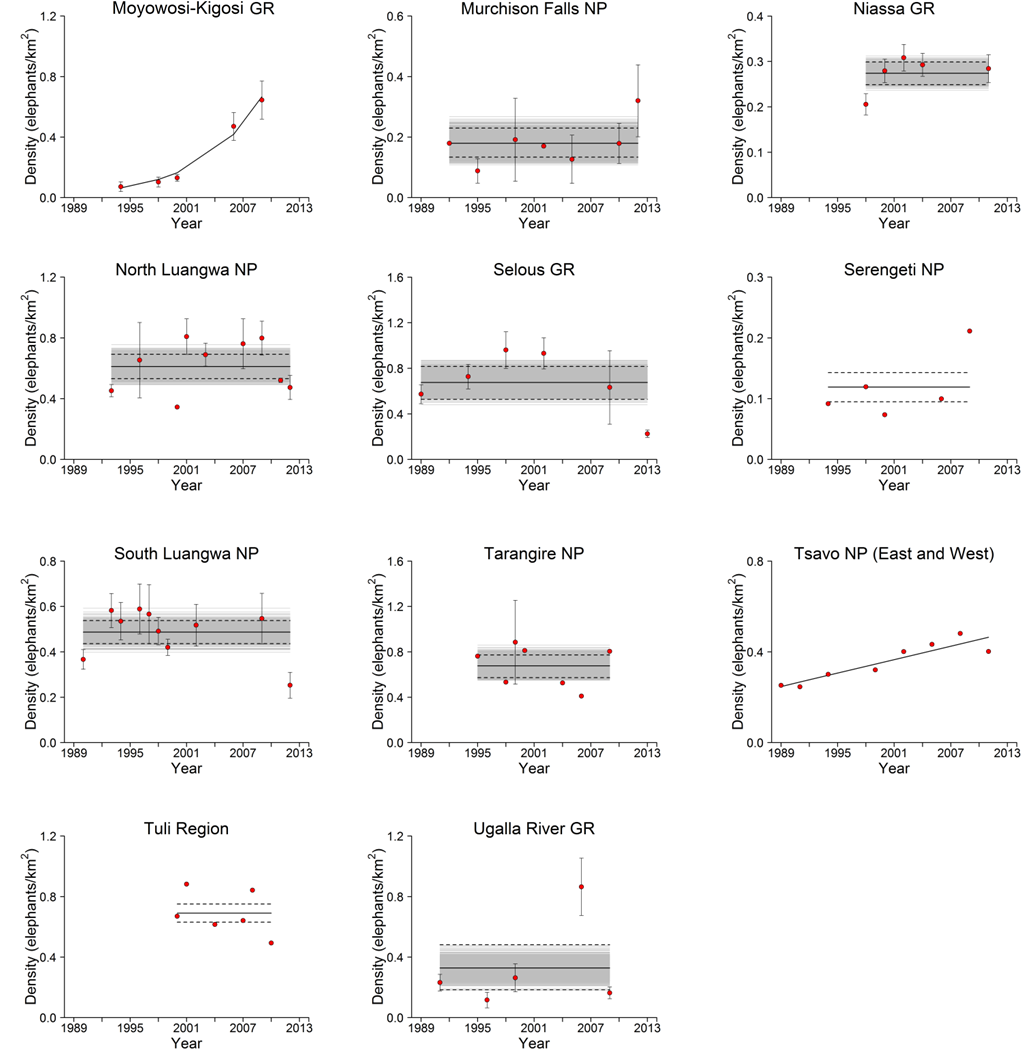


Figure B. Twenty-three time series (error bars=SE) and best-fit population models according to AIC_c_ model selection (details in Tables A and B). Where a null model was selected best, we iterated the time-series 1,000 times based on uncertainty in counts, reperformed model selection to confirm robustness, and refit the best model (grey lines) to incorporate uncertainty into estimates of stable densities (black lines) and their SE’s (dashed lines). Iterations for time series containing only total counts (SE=0) were identical. Where a linear or exponential model was selected, solid black lines represent model fit.


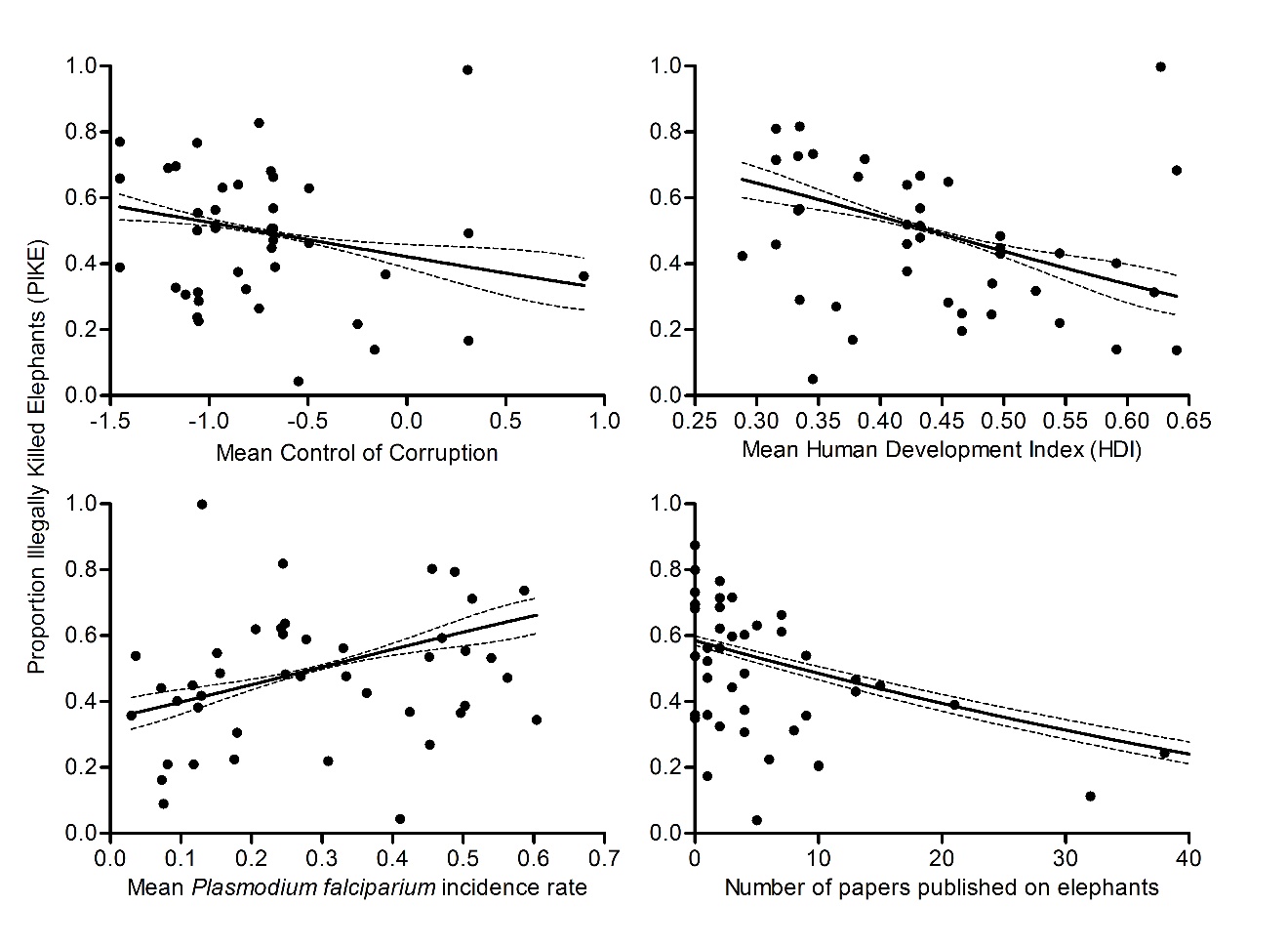


Figure C. Partial residual plots of components of model averaged GLM to explain 43 PIKE values from MIKE sites with variables mean control of corruption (1996-2013), mean Human Development Index (HDI, 1980-2013), mean *Plasmodium falciparum* incidence rate (2000-2013), and number of papers published on elephants (Table E). Model fits (solid lines), SE’s (dotted lines), and partial residuals (points) have been transformed to the response scale of PIKE. Note, an extreme value of papers published on elephants in Kruger National Park (129) and its corresponding partial residual (0.93) was excluded from the plot for ease of visualization.


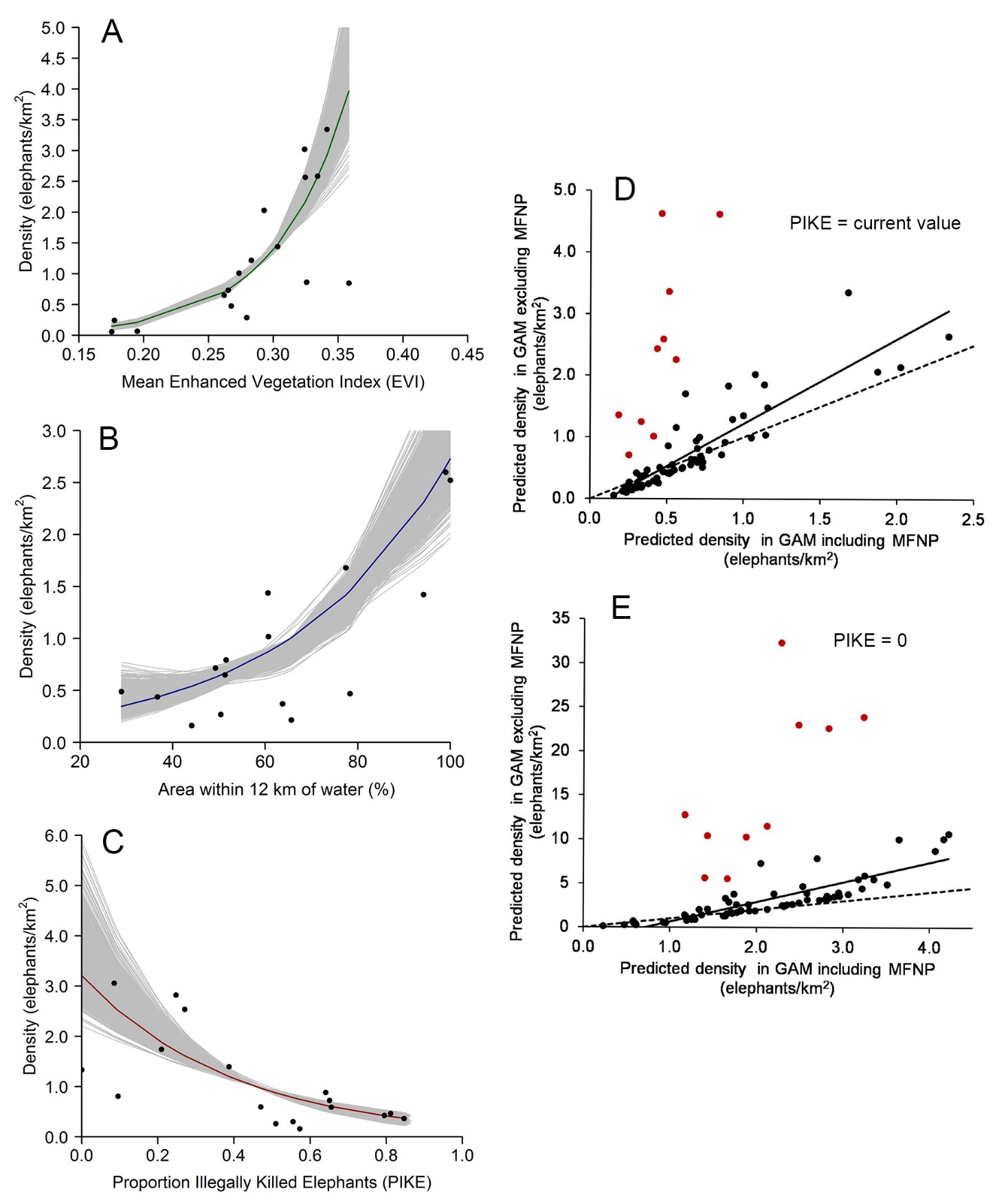


**Figure D.** **Without the influential Murchison Falls National Park data point, the shape of the GAM explaining extracted stable densities from time series of 17 savanna elephant populations differs in shape at high EVI from the model including Murchison Falls (Fig. 1).** The component smooth functions of mean EVI (A), percentage of protected area within 12km of water (B), and PIKE (C) of the selected best GAM are represented by solid lines transformed to the response scale of density (elephants/km^2^). Points represent partial residuals of the 17 extracted densities, and grey lines represent 1,000 iterations to account for uncertainty. When Murchison Falls National Park (MFNP) is excluded, predicted stable density values for the 63 populations that remain within the range of EVI of the training data (black dots) are correlated with the original predicted values for both scenarios as represented by solid black lines: estimates based on current PIKE (panel D, r=0.89, p<0.001) and where PIKE is set to zero (panel E, r=0.86, p<0.001). Dashed lines represent the one-to-one ratio. Predictions for populations with high EVI (red dots represent populations with higher EVI than Selous Game Reserve, the second highest in the dataset after Murchison Falls) reach unreasonably high levels (see recent estimates [12]), especially in the scenario where PIKE is set to zero (E). Localized densities > 4 elephants/km^2^ have been recorded in the past [33], but the highest density from recent estimates is 2.7 elephants/km^2^ in Chirisa Safari Area (Table F). Therefore, Murchison Falls is retained in the model used throughout the rest of the text.


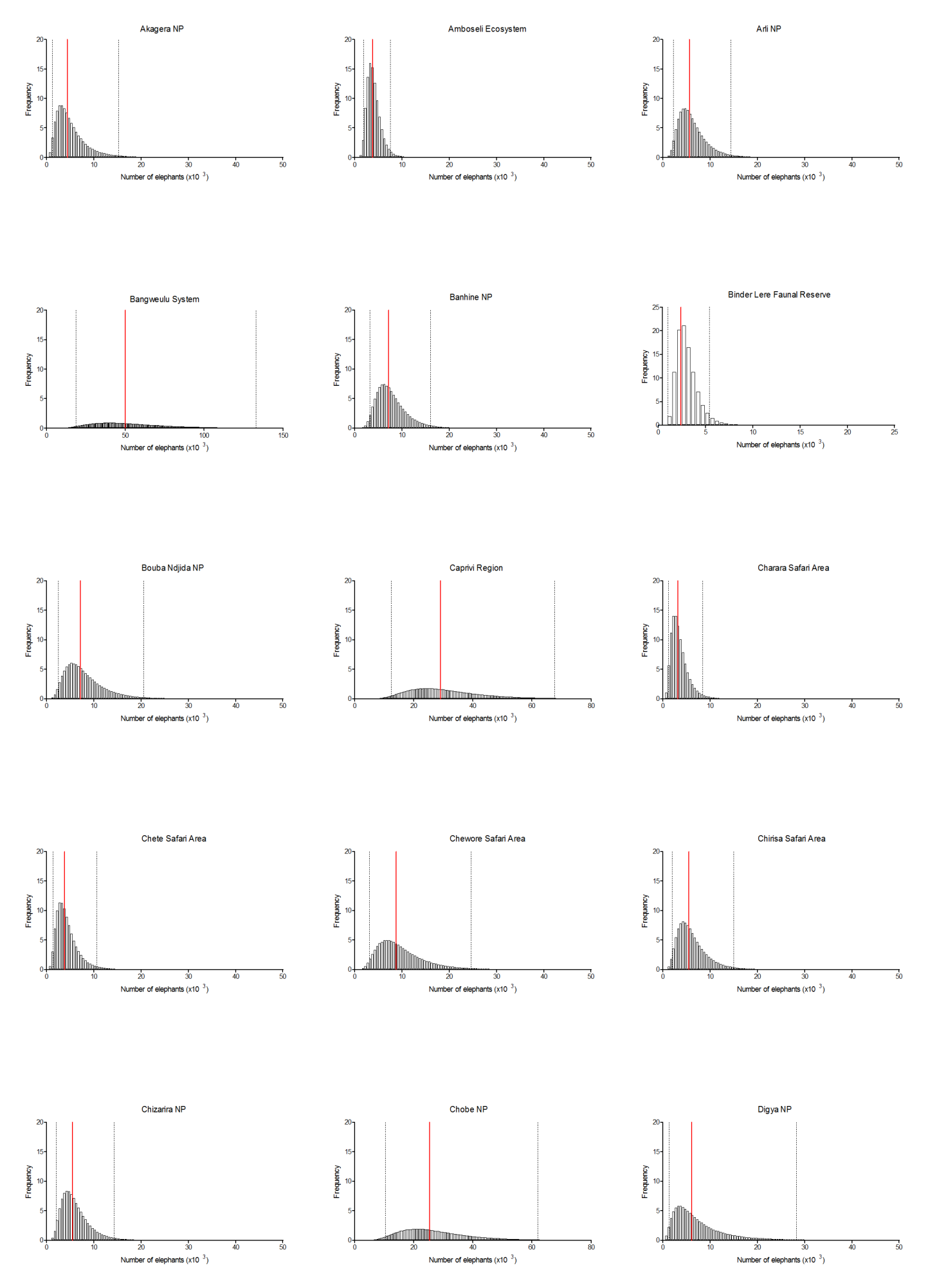


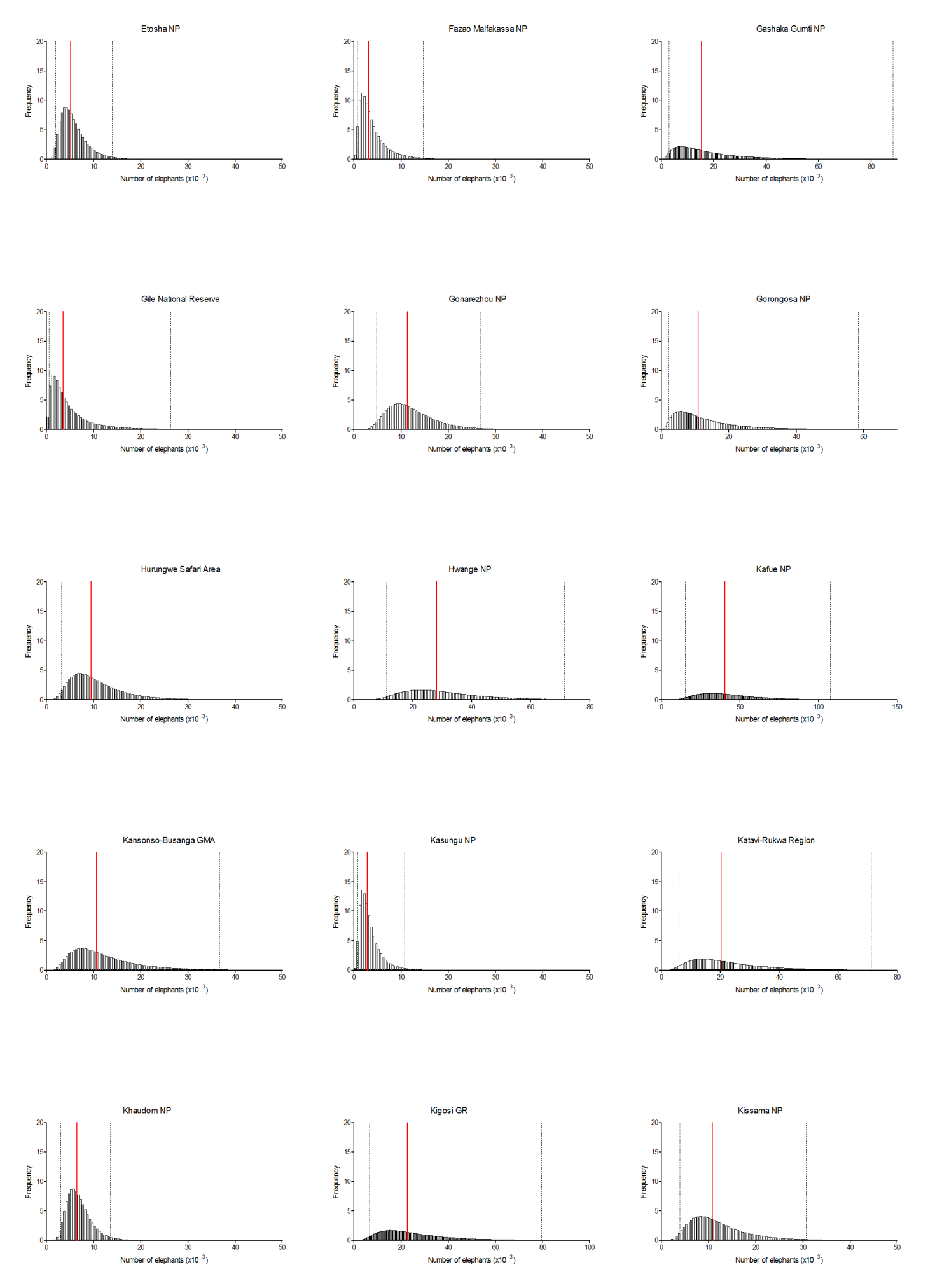


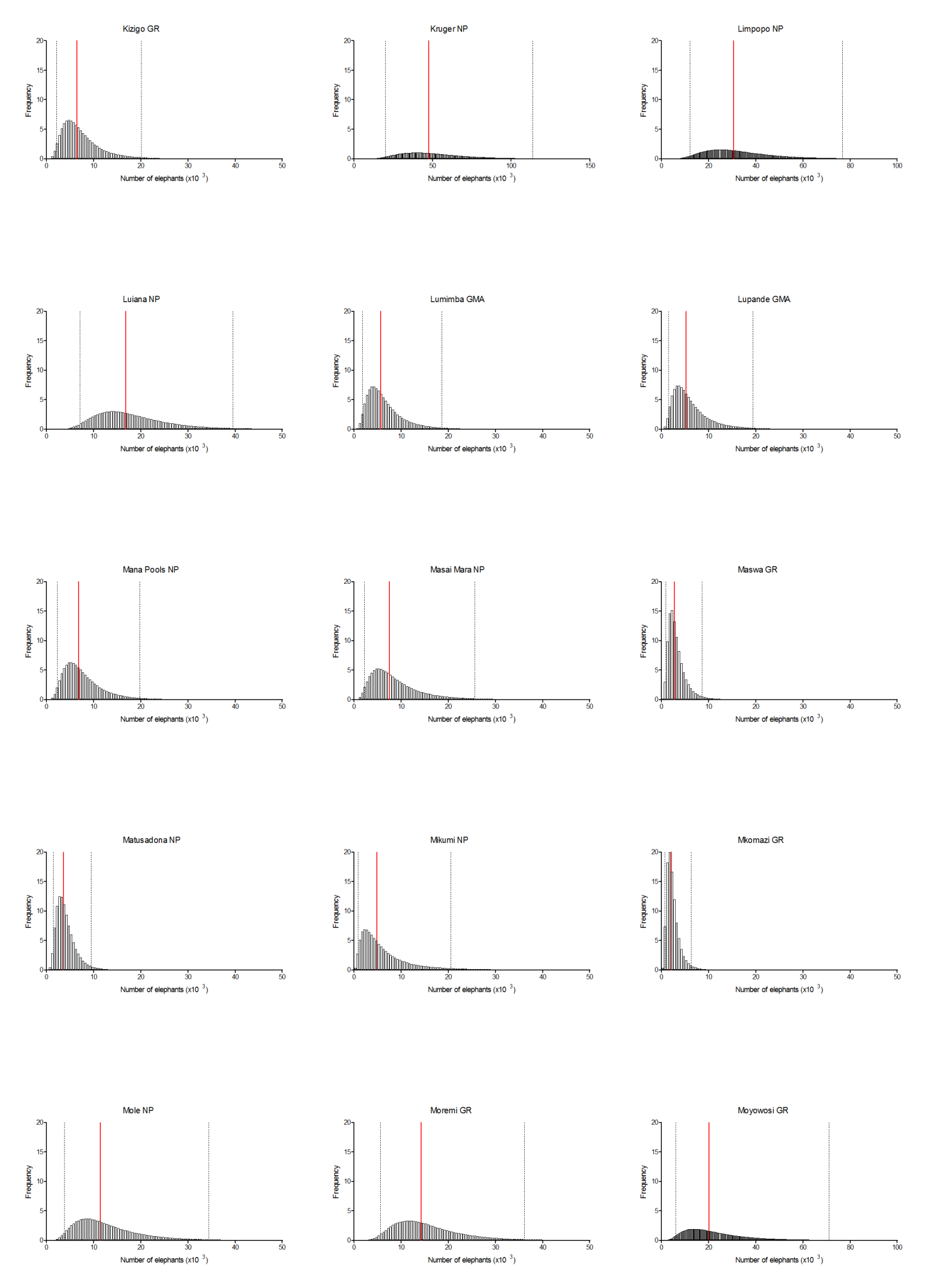


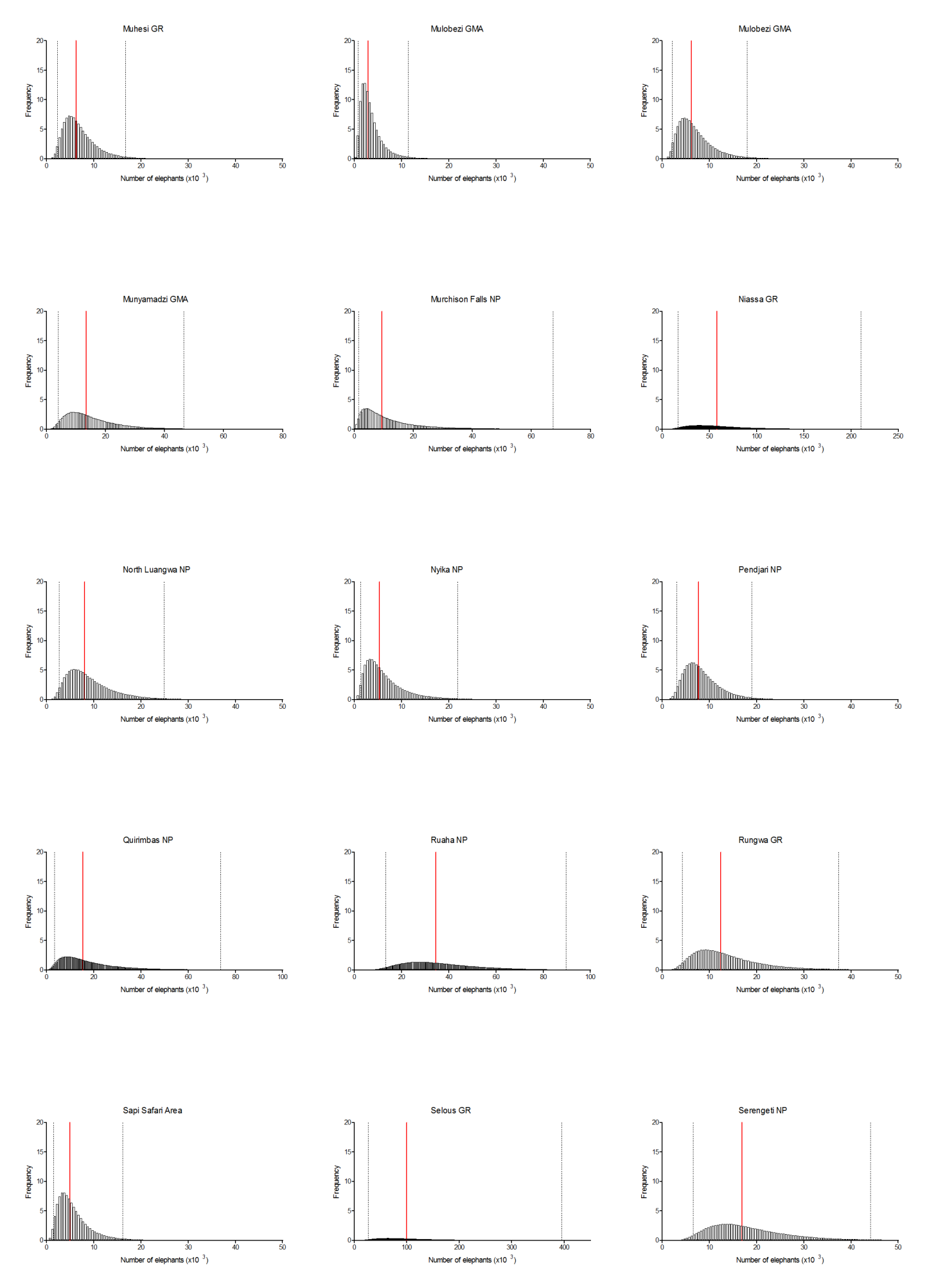


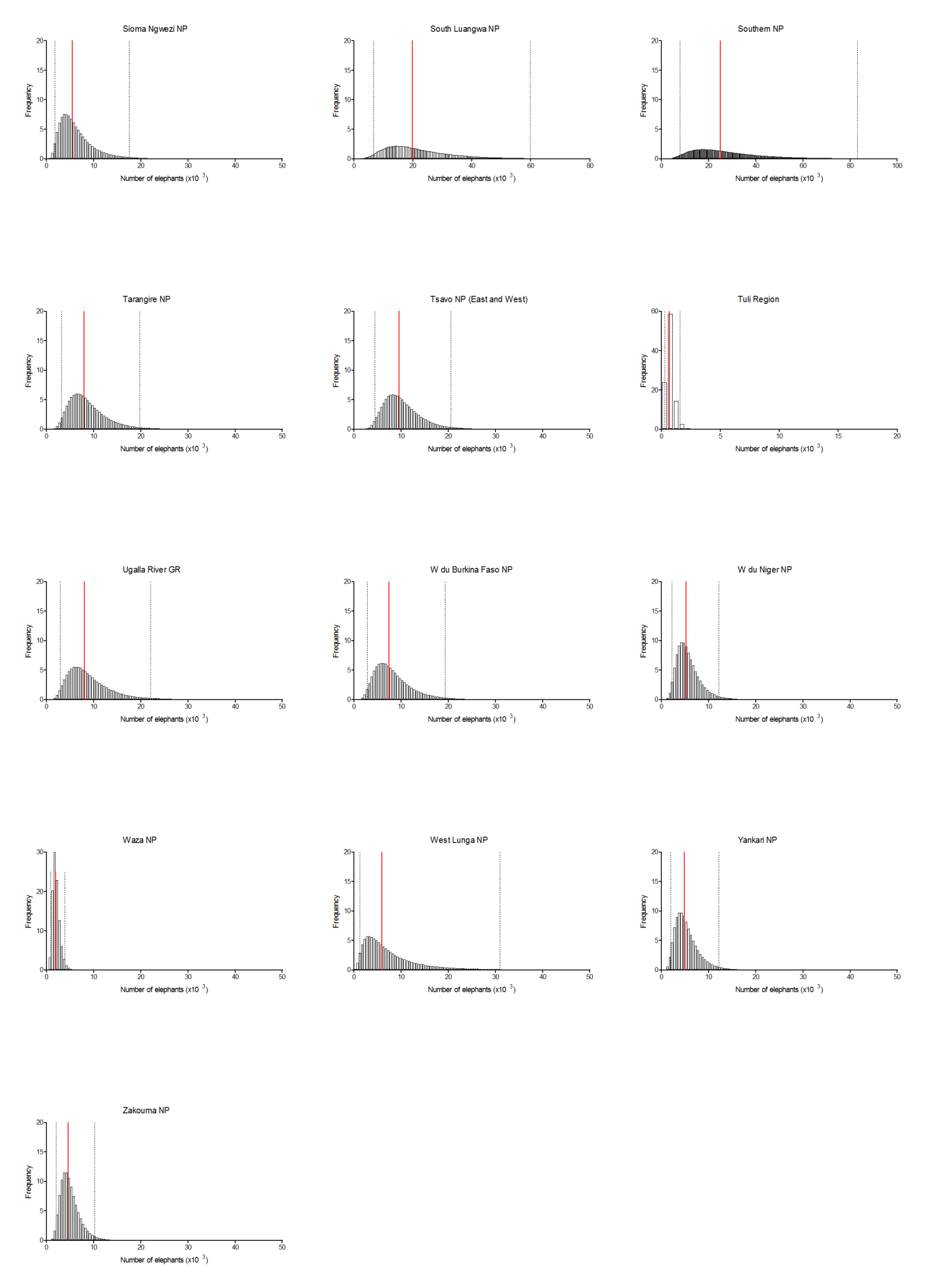


Figure E. Histogram of ecological benchmark estimates for each of 73 protected areas recalculated for each of 1x10^6 runs from the Monte Carlo simulation to incorporate uncertainty. Median ecological benchmark shown as red line and dashed lines represent 95% prediction intervals.


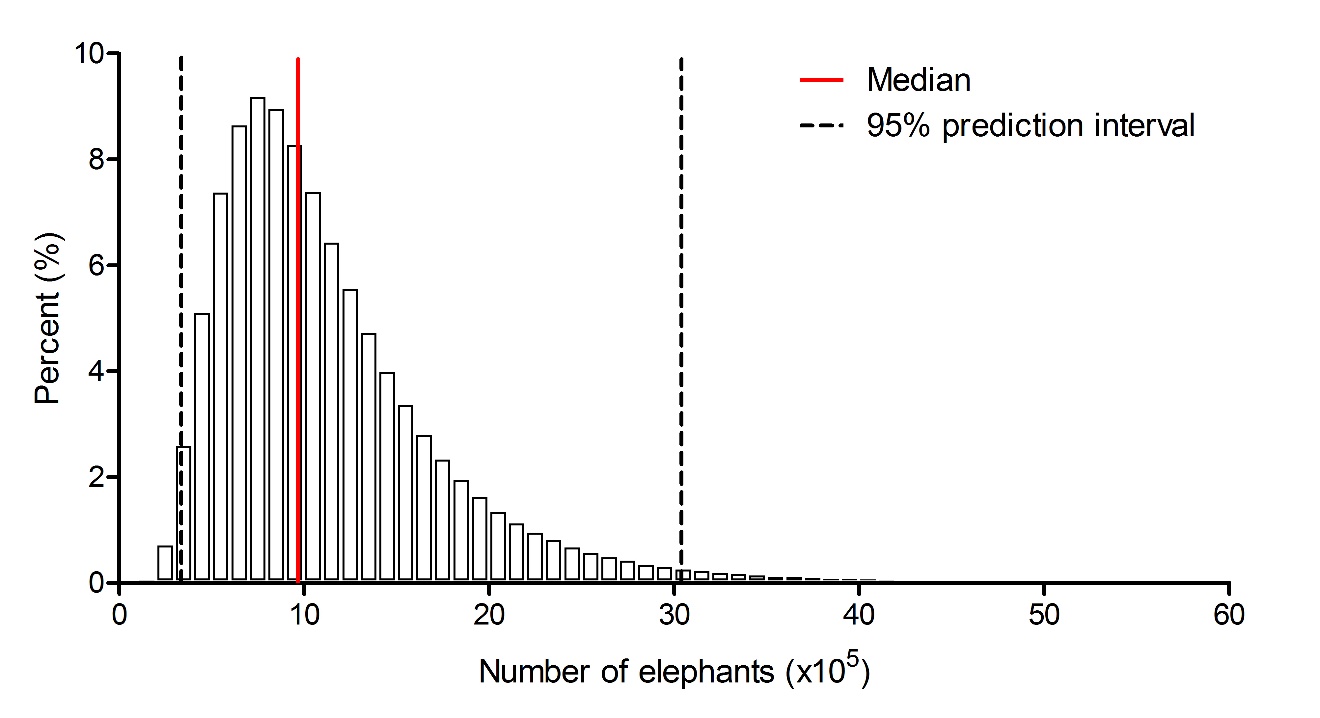


Figure F. Histogram of cumulative ecological benchmark across 73 protected areas recalculated for each of 1x10^6 runs from the Monte Carlo simulation to incorporate uncertainty. Median ecological benchmark=967,014, lower 95% prediction level=336,059 and upper 95% prediction level= 3,040,724.

Table A. Alternative candidate models to describe the population dynamics of 23 time-series populations. Candidate models with the lowest AIC_c_ are indicated in bold. Dashes show where candidate models failed to converge after a maximum of 100,000 iterations. “Par” is number of parameters. Models for logistic and Gompertz dynamics were respectively: *y = a / (1 + exp((4u / a) * (i – x) + 2))* and *y = a * exp(-exp(((u * exp(1)) / a) * (i – x) + 1))*, where *y* = density at year *x*, *a* = asymptote, and *u* and *i* are parameters specific for the functions].

| Protected Area | Candidate model | Par | AIC_c_ | ΔAIC_c_ | AIC_c_ (*wi*) | R^2^ |
| --- | --- | --- | --- | --- | --- | --- |
| Amboseli Ecosystem | **Null (no trend)** | **2** | **-38.21** | **0.00** | **0.81** | **0.00** |
|  | Linear | 3 | -33.78 | 4.43 | 0.09 | 0.31 |
|  | Exponential | 3 | -34.00 | 4.21 | 0.10 | 0.33 |
|  | Logistic | 4 | -20.00 | 18.21 | 0.00 | 0.33 |
|  | Gompertz | 4 | -19.97 | 18.24 | 0.00 | 0.33 |
| Arli National Park | **Null (no trend)** | **2** | **-14.00** | **0.00** | **0.95** | **0.00** |
|  | Linear | 3 | -6.36 | 7.64 | 0.02 | 0.32 |
|  | Exponential | 3 | -6.78 | 7.22 | 0.03 | 0.37 |
|  | Logistic | 4 | 22.79 | 36.79 | 0.00 | 0.41 |
|  | Gompertz | 4 | 22.91 | 36.91 | 0.00 | 0.40 |
| Caprivi Region | **Null (no trend)** | **2** | **-28.54** | **0.00** | **0.58** | **0.00** |
|  | Linear | 3 | -25.61 | 2.93 | 0.13 | 0.09 |
|  | Exponential | 3 | -24.97 | 3.57 | 0.10 | 0.03 |
|  | Logistic | 4 | -24.86 | 3.68 | 0.09 | 0.39 |
|  | Gompertz | 4 | -24.86 | 3.68 | 0.09 | 0.39 |
| Chobe National Park | **Null (no trend)** | **2** | **-2.74** | **0.00** | **0.37** | **0.00** |
|  | Linear | 3 | -0.49 | 2.25 | 0.12 | 0.18 |
|  | Exponential | 3 | 0.35 | 3.08 | 0.08 | 0.11 |
|  | Logistic | 4 | -1.77 | 0.96 | 0.23 | 0.61 |
|  | Gompertz | 4 | -1.65 | 1.09 | 0.2 | 0.60 |
| Etosha National Park | **Null (no trend)** | **2** | **-26.40** | **0.00** | **1.00** | **0.00** |
|  | Linear | 3 | -8.31 | 18.09 | 0.00 | 0.32 |
|  | Exponential | 3 | -8.51 | 17.89 | 0.00 | 0.34 |
|  | Logistic | — | — | — | — | — |
|  | Gompertz | — | — | — | — | — |
| Gonarezhou National Park | Null (no trend) | 2 | -5.80 | 7.54 | 0.02 | 0.00 |
|  | Linear | 3 | -9.93 | 3.41 | 0.15 | 0.70 |
|  | **Exponential** | **3** | **-13.34** | **0.00** | **0.82** | **0.81** |
|  | Logistic | 4 | -4.32 | 9.02 | 0.01 | 0.81 |
|  | Gompertz | 4 | -3.87 | 9.46 | 0.01 | 0.80 |
| Hwange National Park | **Null (no trend)** | **2** | **-19.11** | **0.00** | **0.36** | **0.00** |
|  | Linear | 3 | -18.70 | 0.41 | 0.29 | 0.21 |
|  | Exponential | 3 | -18.63 | 0.47 | 0.28 | 0.21 |
|  | Logistic | 4 | -14.44 | 4.67 | 0.03 | 0.21 |
|  | Gompertz | 4 | -14.43 | 4.68 | 0.03 | 0.21 |
| Kafue National Park | Null (no trend) | 2 | -51.84 | 4.60 | 0.06 | 0.00 |
|  | Linear | 3 | -54.75 | 1.69 | 0.26 | 0.51 |
|  | **Exponential** | **3** | **-56.44** | **0.00** | **0.61** | **0.59** |
|  | Logistic | 4 | -50.88 | 5.55 | 0.04 | 0.61 |
|  | Gompertz | 4 | -50.62 | 5.82 | 0.03 | 0.60 |
| Katavi-Rukwa Region | **Null (no trend)** | **2** | **-24.16** | **0.00** | **0.94** | **0.00** |
|  | Linear | 3 | -17.39 | 6.77 | 0.03 | 0.42 |
|  | Exponential | 3 | -17.37 | 6.79 | 0.03 | 0.41 |
|  | Logistic | 4 | 12.60 | 36.76 | 0.00 | 0.42 |
|  | Gompertz | 4 | 12.61 | 36.76 | 0.00 | 0.42 |
| Kruger National Park | Null (no trend) | 2 | -70.61 | 52.12 | 0.00 | 0.00 |
|  | Linear | 3 | -120.37 | 2.37 | 0.18 | 0.95 |
|  | **Exponential** | **3** | **-122.73** | **0.00** | 0.59 | **0.95** |
|  | Logistic | 4 | -119.45 | 3.29 | 0.11 | 0.95 |
|  | Gompertz | 4 | -119.42 | 3.32 | 0.11 | 0.95 |
| Lupande Game Management Area | **Null (no trend)** | **2** | **-12.41** | **0.00** | **0.67** | **0.00** |
|  | Linear | 3 | -9.27 | 3.14 | 0.14 | 0.26 |
|  | Exponential | 3 | -9.83 | 2.58 | 0.18 | 0.31 |
|  | Logistic | 4 | -1.18 | 11.23 | 0.00 | 0.37 |
|  | Gompertz | 4 | -0.97 | 11.44 | 0.00 | 0.35 |
| Moremi Game Reserve | **Null (no trend)** | **2** | **-3.24** | **0.00** | **0.94** | **0.00** |
|  | Linear | 3 | 3.74 | 6.98 | 0.03 | 0.00 |
|  | Exponential | 4 | 3.74 | 6.98 | 0.03 | 0.00 |
|  | Logistic | 4 | 17.74 | 20.98 | 0.00 | 0.00 |
|  | Gompertz | 3 | 17.74 | 20.98 | 0.00 | 0.00 |
| Moyowosi Game Reserve | Null (no trend) | 2 | -4.67 | 0.15 | 0.48 | 0.00 |
|  | Linear | 3 | 2.81 | 7.62 | 0.01 | 0.92 |
|  | **Exponential** | **3** | **-4.81** | **0.00** | **0.51** | **0.98** |
|  | Logistic | — | — | — | — | — |
|  | Gompertz | — | — | — | — | — |
| Murchison Falls National Park | **Null (no trend)** | **2** | **-30.91** | **0.00** | **0.78** | **0.00** |
|  | Linear | 3 | -26.74 | 4.17 | 0.10 | 0.33 |
|  | Exponential | 3 | -27.28 | 3.63 | 0.13 | 0.38 |
|  | Logistic | 4 | -13.32 | 17.59 | 0.00 | 0.39 |
|  | Gompertz | 4 | -13.23 | 17.68 | 0.00 | 0.38 |
| Niassa Game Reserve | **Null (no trend)** | **2** | **-23.34** | **0.00** | **1.00** | **0.00** |
|  | Linear | 3 | -4.73 | 18.60 | 0.00 | 0.24 |
|  | Exponential | 3 | -4.61 | 18.73 | 0.00 | 0.22 |
|  | Logistic | — | — | — | — | — |
|  | Gompertz | — | — | — | — | — |
| North Luangwa National Park | **Null (no trend)** | **2** | **-27.08** | **0.00** | **0.83** | **0.00** |
|  | Linear | 3 | -22.51 | 4.56 | 0.08 | 0.03 |
|  | Exponential | 3 | -22.49 | 4.59 | 0.08 | 0.02 |
|  | Logistic | 4 | -16.27 | 10.80 | 0.00 | 0.12 |
|  | Gompertz | 4 | -16.27 | 10.80 | 0.00 | 0.12 |
| Selous Game Reserve | **Null (no trend)** | **2** | **-8.81** | **0.00** | **0.98** | **0.00** |
|  | Linear | 3 | -0.16 | 8.66 | 0.01 | 0.20 |
|  | Exponential | 3 | 0.55 | 9.36 | 0.01 | 0.10 |
|  | Logistic | 4 | 23.48 | 32.29 | 0.00 | 0.72 |
|  | Gompertz | 4 | 23.56 | 32.37 | 0.00 | 0.72 |
| Serengeti National Park | **Null (no trend)** | **2** | **-20.30** | **0.00** | **1.00** | **0.00** |
|  | Linear | 3 | -3.24 | 17.06 | 0.00 | 0.44 |
|  | Exponential | 3 | -3.80 | 16.50 | 0.00 | 0.50 |
|  | Logistic | — | — | — | — | — |
|  | Gompertz | — | — | — | — | — |
| South Luangwa National Park | **Null (no trend)** | **2** | **-39.72** | **0.00** | **0.44** | **0.00** |
|  | Linear | 3 | -36.90 | 2.77 | 0.11 | 0.14 |
|  | Exponential | 3 | -36.75 | 2.97 | 0.10 | 0.12 |
|  | Logistic | 4 | -37.90 | 1.82 | 0.18 | 0.57 |
|  | Gompertz | 4 | -37.90 | 1.82 | 0.18 | 0.57 |
| Tarangire National Park | **Null (no trend)** | **2** | **-17.92** | **0.00** | **0.93** | **0.00** |
|  | Linear | 3 | -11.41 | 6.52 | 0.04 | 0.07 |
|  | Exponential | 3 | -11.44 | 6.48 | 0.04 | 0.07 |
|  | Logistic | 4 | 2.56 | 20.49 | 0.00 | 0.07 |
|  | Gompertz | 4 | 2.57 | 20.49 | 0.00 | 0.07 |
| Tsavo National Park (East and West) | Null (no trend) | 2 | -33.78 | 8.86 | 0.01 | 0.00 |
|  | **Linear** | **3** | **-42.64** | **0.00** | **0.65** | **0.84** |
|  | Exponential | 3 | -41.21 | 1.42 | 0.32 | 0.80 |
|  | Logistic | 4 | -34.88 | 7.76 | 0.01 | 0.87 |
|  | Gompertz | 4 | -34.78 | 8.04 | 0.0 | 0.86 |
| Tuli Region | **Null (no trend)** | **2** | **-16.15** | **0.00** | **0.98** | **0.00** |
|  | Linear | 3 | -7.29 | 8.85 | 0.01 | 0.17 |
|  | Exponential | 3 | -7.25 | 8.90 | 0.01 | 0.17 |
|  | Logistic | 4 | 20.40 | 36.54 | 0.00 | 0.44 |
|  | Gompertz | 4 | 20.40 | 36.54 | 0.00 | 0.44 |
| Ugalla Game Reserve | **Null (no trend)** | **2** | **-2.97** | **0.00** | **1.00** | **0.00** |
|  | Linear | 3 | 16.19 | 19.16 | 0.00 | 0.15 |
|  | Exponential | 3 | 16.31 | 19.28 | 0.00 | 0.13 |
|  | Logistic | — | — | — | — | — |
|  | Gompertz | — | — | — | — | — |

Table B. Summary information on time series for 23 populations plotted in Figure B, best fit model from Table A, and extracted stable density with SE calculated after fitting best model to 1,000 simulations of count data based on uncertainty reported for counts. Stability of null best fit model is the percent of those 1,000 simulations where null was selected best (* indicates time-series including only total counts where SE=0, thus, all 1,000 simulations were identical). For each time series, “period” indicates the spread of years covered by “n” population estimates.

| Protected Area | Period | n | Best fit model | Extracted stable density ± S.E. | Stability of null best fit model (%) |
| --- | --- | --- | --- | --- | --- |
| Amboseli Ecosystem | 2000 - 2013 | 7 | Null (no trend) | 0.209 ± 0.016 | 100* |
| Arli National Park | 1998 - 2013 | 6 | Null (no trend) | 0.415 ± 0.117 | 99.7 |
| Caprivi Region | 1989 - 2013 | 11 | Null (no trend) | 0.614 ± 0.097 | 96.7 |
| Chobe National Park | 1992 - 2011 | 10 | Null (no trend) | 2.587 ± 0.287 | 91.2 |
| Etosha National Park | 1989 - 2004 | 5 | Null (no trend) | 0.095 ± 0.016 | 100 |
| Gonarezhou National Park | 1989 - 2013 | 8 | Exponential | — | — |
| Hwange National Park | 1989 - 2007 | 13 | Null (no trend) | 2.091 ± 0.142 | 72.5 |
| Kafue National Park | 1991 - 2011 | 10 | Exponential | — | — |
| Katavi-Rukwa Region | 1991 - 2009 | 6 | Null (no trend) | 0.493 ± 0.098 | 97.1 |
| Kruger National Park | 1995 - 2012 | 18 | Exponential | — | — |
| Lupande Game Management Area | 1989 - 2012 | 8 | Null (no trend) | 0.408 ± 0.143 | 100 |
| Moremi Game Reserve | 1996 - 2010 | 7 | Null (no trend) | 1.922 ± 0.326 | 99.9 |
| Moyowosi Game Reserve | 1994 - 2009 | 5 | Exponential | — | — |
| Murchison Falls National Park | 1992 - 2012 | 7 | Null (no trend) | 0.182 ± 0.048 | 96.8 |
| Niassa Game Reserve | 1998 - 2011 | 5 | Null (no trend) | 0.274 ± 0.025 | 99.9 |
| North Luangwa National Park | 1993 - 2012 | 9 | Null (no trend) | 0.612 ± 0.080 | 100 |
| Selous Game Reserve | 1989 - 2013 | 6 | Null (no trend) | 0.672 ± 0.144 | 100 |
| Serengeti National Park | 1994 - 2009 | 5 | Null (no trend) | 0.119 ± 0.024 | 100* |
| South Luangwa National Park | 1990 - 2012 | 10 | Null (no trend) | 0.487 ± 0.051 | 100 |
| Tarangire National Park | 1995 - 2009 | 7 | Null (no trend) | 0.673 ± 0.100 | 100 |
| Tsavo National Park (East and West) | 1989 - 2011 | 8 | Linear | — | — |
| Tuli Region | 2000 - 2010 | 6 | Null (no trend) | 0.691 ± 0.060 | 100* |
| Ugalla Game Reserve | 1991 - 2009 | 5 | Null (no trend) | 0.332 ± 0.149 | 100 |

**Table C. Summary information for 43 MIKE sites and the explanatory variables used to explain PIKE within a quasi-binomial generalized linear modelling framework.** Note: CAR = Central African Republic; DRC = Democratic Republic of the Congo; S = savanna; F = forest; B = both savanna and forest; *con_of_corr* = mean Control of Corruption (1996-2013); *HDI* = mean Human Development Index (1980-2013); *malaria* = mean *Plasmodium falciparum* incidence rate (2000-2013); *tree_cover* = mean percentage tree cover (2000-2013); *ele_interest* = scientific interest in elephants.

| MIKE site | Country | Total carcasses | Poached carcasses | PIKE | Sub-species | Human density (/km^2^) | Area (km^2^) | *HDI* | *con_of_corr* | *malaria* | *tree_cover* | *ele_interest* |
| --- | --- | --- | --- | --- | --- | --- | --- | --- | --- | --- | --- | --- |
| Babile Elephant Sanctuary | Ethiopia | 34 | 26 | 0.76 | S | 36.22 | 6949 | 0.39 | -0.69 | 0.04 | 6.02 | 3 |
| Bangassou Classified Forest | CAR | 59 | 58 | 0.98 | F | 2.83 | 12002 | 0.33 | -1.06 | 0.46 | 58.14 | 0 |
| Boumba Bek National Park | Cameroon | 80 | 45 | 0.56 | F | 3.14 | 2365 | 0.47 | -1.05 | 0.50 | 76.53 | 4 |
| Cabora Basa | Mozambique | 108 | 91 | 0.84 | S | 9.00 | 2934 | 0.33 | -0.50 | 0.24 | 23.24 | 2 |
| Caprivi Region | Namibia | 225 | 87 | 0.39 | S | 3.13 | 3196 | 0.59 | 0.31 | 0.07 | 6.70 | 4 |
| Chewore Safari Area | Zimbabwe | 399 | 154 | 0.39 | S | 15.35 | 3400 | 0.45 | -1.17 | 0.08 | 11.05 | 1 |
| Chobe National Park | Botswana | 1555 | 133 | 0.09 | S | 0.88 | 10596 | 0.62 | 0.89 | 0.03 | 4.47 | 38 |
| Dzanga-Sangha Protected Area | CAR | 134 | 82 | 0.61 | F | 3.09 | 4647 | 0.33 | -1.06 | 0.45 | 75.68 | 6 |
| Etosha National Park | Namibia | 212 | 0 | 0.00 | S | 0.12 | 22942 | 0.59 | 0.31 | 0.07 | 1.67 | 32 |
| Garamba National Park | DRC | 670 | 617 | 0.92 | B | 7.99 | 14835 | 0.32 | -1.45 | 0.59 | 22.37 | 5 |
| Gash-Setit | Eritrea | 39 | 7 | 0.18 | S | 22.63 | 5275 | 0.38 | -0.16 | 0.08 | 37.57 | 1 |
| Gourma | Mali | 62 | 13 | 0.21 | S | 3.56 | 28090 | 0.35 | -0.55 | 0.41 | 2.89 | 5 |
| Katavi Rukwa | Tanzania | 159 | 129 | 0.81 | S | 2.30 | 8356 | 0.43 | -0.68 | 0.25 | 48.70 | 2 |
| Kruger National Park | South Africa | 285 | 9 | 0.03 | S | 26.17 | 19044 | 0.63 | 0.31 | 0.13 | 26.33 | 129 |
| Lope National Park | Gabon | 79 | 14 | 0.18 | F | 0.83 | 4934 | 0.64 | -0.75 | 0.18 | 10.13 | 2 |
| Meru National Park | Kenya | 411 | 240 | 0.58 | S | 8.02 | 4906 | 0.50 | -0.97 | 0.12 | 5.51 | 3 |
| Minkebe National Park | Gabon | 160 | 140 | 0.88 | F | 0.93 | 7539 | 0.64 | -0.75 | 0.24 | 4.95 | 0 |
| Mole National Park | Ghana | 23 | 14 | 0.61 | S | 1.07 | 4494 | 0.53 | -0.11 | 0.54 | 5.10 | 1 |
| Mount Elgon Forest Reserve | Kenya | 86 | 54 | 0.63 | S | 77.21 | 595 | 0.50 | -0.97 | 0.27 | 47.66 | 1 |
| Murchison Falls National Park | Uganda | 94 | 72 | 0.77 | S | 12.16 | 5249 | 0.42 | -0.85 | 0.15 | 55.24 | 0 |
| Nazinga Game Ranch | Burkina Faso | 26 | 15 | 0.58 | S | 7.61 | 922 | 0.36 | -0.25 | 0.60 | 76.53 | 10 |
| Niassa Game Reserve | Mozambique | 195 | 155 | 0.79 | S | 2.39 | 42523 | 0.33 | -0.50 | 0.45 | 8.58 | 1 |
| Nouabale Ndoki National Park | Congo | 84 | 35 | 0.42 | F | 0.44 | 4074 | 0.55 | -1.06 | 0.42 | 22.20 | 8 |
| Nyaminyami district | Zimbabwe | 147 | 120 | 0.82 | S | 5.66 | 2775 | 0.45 | -1.17 | 0.21 | 6.78 | 0 |
| Odzala-Kokoua National Park | Congo | 347 | 206 | 0.59 | F | 0.53 | 13762 | 0.55 | -1.06 | 0.33 | 72.91 | 9 |
| Okapi Wildlife Reserve | DRC | 192 | 187 | 0.97 | F | 11.82 | 14062 | 0.32 | -1.45 | 0.49 | 77.70 | 2 |
| Parc W du Niger | Niger | 31 | 18 | 0.58 | S | 0.38 | 2212 | 0.29 | -0.81 | 0.12 | 3.80 | 0 |
| Park W du Benin | Benin | 26 | 20 | 0.77 | S | 7.78 | 5784 | 0.42 | -0.68 | 0.47 | 5.06 | 2 |
| Pendjari Biosphere Reserve | Benin | 88 | 64 | 0.73 | S | 7.89 | 2827 | 0.42 | -0.68 | 0.50 | 5.23 | 4 |
| Queen Elizabeth Protected Area | Uganda | 156 | 70 | 0.45 | F | 10.58 | 2496 | 0.42 | -0.85 | 0.18 | 34.88 | 9 |
| Ruaha Rungwa Kizigo Muhesi | Tanzania | 327 | 221 | 0.68 | S | 0.71 | 25892 | 0.43 | -0.68 | 0.16 | 10.88 | 2 |
| Salonga National Park | DRC | 137 | 110 | 0.80 | B | 3.46 | 33370 | 0.32 | -1.45 | 0.56 | 78.29 | 4 |
| Samburu-Laikipia | Kenya | 2428 | 1024 | 0.42 | S | 28.37 | 32348 | 0.50 | -0.97 | 0.09 | 5.94 | 15 |
| Sangba Pilot Zone | CAR | 46 | 36 | 0.78 | S | 0.55 | 10600 | 0.33 | -1.06 | 0.33 | 19.40 | 0 |
| Selous Mikumi | Tanzania | 1048 | 600 | 0.57 | S | 1.00 | 51027 | 0.43 | -0.68 | 0.25 | 20.34 | 13 |
| South Luangwa National Park | Zambia | 310 | 172 | 0.55 | S | 2.45 | 8931 | 0.49 | -0.67 | 0.36 | 17.79 | 3 |
| Tarangire Manyara | Tanzania | 117 | 55 | 0.47 | S | 19.80 | 9463 | 0.43 | -0.68 | 0.12 | 6.46 | 13 |
| Tsavo Conservation Area | Kenya | 1651 | 632 | 0.38 | S | 8.59 | 37398 | 0.50 | -0.97 | 0.13 | 3.34 | 21 |
| Virunga National Park | DRC | 166 | 140 | 0.84 | F | 41.20 | 7770 | 0.32 | -1.45 | 0.28 | 38.22 | 7 |
| Waza National Park | Cameroon | 23 | 7 | 0.30 | S | 13.96 | 1407 | 0.47 | -1.05 | 0.31 | 7.55 | 10 |
| Yankari National Park | Nigeria | 50 | 30 | 0.60 | S | 26.45 | 2387 | 0.49 | -1.12 | 0.50 | 4.42 | 0 |
| Zakouma National Park | Chad | 453 | 384 | 0.85 | S | 3.67 | 4971 | 0.35 | -1.21 | 0.24 | 6.02 | 7 |
| Ziama Classified Forest | Guinea | 28 | 25 | 0.89 | F | 25.72 | 915 | 0.38 | -0.93 | 0.51 | 66.13 | 0 |

Table D. Summary statistics and variables included in most likely (ΔQAICc<2) quasi-binomial generalized linear models to explain PIKE values for 43 MIKE sites across Africa and final predictive average model used to generate PIKE estimates for non-MIKE sites. Note: *con_of_corr* = mean Control of Corruption (1996-2013), *HDI* = mean Human Development Index (1980-2013), *malaria* = mean *Plasmodium falciparum* incidence rate (2000-2013), *ele_interest* = scientific interest in elephants. * = 0.05< *p* < 0.10, ** = 0.01< *p* < 0.05, *** = *p* < 0.01.

|  |  |  |  |  |  | Country-level covariates | | Site-level covariates | |
| --- | --- | --- | --- | --- | --- | --- | --- | --- | --- |
| Rank | R^2^ | QAICc | ΔQAICc | QAICc_(_*_wi_*_)_ | Intercept | *con_of_corr* | *HDI* | *malaria* | *ele_interest* |
| 1 | 0.83 | 60.32 | 0.00 | 0.21 | 1.48 ± 0.88 | -0.42 ± 0.22* | -3.76 ± 1.51** | 2.20 ± 0.79*** | -0.04 ± 0.01*** |
| 2 | 0.81 | 61.00 | 0.68 | 0.15 | 2.46 ± 0.78*** | — | -4.88 ± 1.50*** | 2.09 ± 0.85** | -0.04 ± 0.01*** |
| Average model (Predictive model) | | | | | 1.89 ± 0.97* | -0.42 ± 0.22* | -4.23 ± 1.60** | 2.16 ± 0.82** | -0.04 ± 0.01*** |
| Relative variable importance | | | |  |  | 0.58 | 1.00 | 1.00 | 1.00 |

Table E. Selection parameters of candidate generalized additive models explaining variation in extracted stable population size for 18 populations. For each model, degrees of freedom (df), AIC corrected for small sample size (AIC_c_), change in AIC_c_ (ΔAIC_c_), AIC_c_ weight (AIC_c_ (wi)), deviance explained (R^2^), adjusted deviance explained (R^2^_adj_), correlation coefficient (COR), cross-validation correlation coefficient (cvCOR), Wilmott’s index of agreement (D) and mean bias error (MBE) are shown. The best fit model, shown in bold, including *EVI*, the mean EVI (2000-2013); *prop_12_water*, the proportion of protected area within 12km of water; and *PIKE*, Percentage Illegally Killed Elephants. The model explained 84% of variation in stable population size, with each included term improving the model by more than chance (R^2^_adj_ = 0.83). Under LOOCV, the model’s predictive performance was relatively stable and accurate (COR = 0.95; cvCOR = 0.83) and agreed well with the observed values (D = 0.88). The model only had a slight bias towards underestimating elephant numbers (MBE = -18 elephants).

| Candidate models | df | AICc | ΔAIC_c_ | AIC_c_ (*wi*) | R^2^ | R^2^ _adj_ | COR | cvCOR | D | MBE |
| --- | --- | --- | --- | --- | --- | --- | --- | --- | --- | --- |
| ***EVI* + *prop_12_water* + *PIKE*** | **6** | **344.80** | **0.00** | **0.93** | **0.84** | **0.83** | **0.95** | **0.83** | **0.88** | **-18** |
| *EVI* + *prop_12_water* | 5 | 350.00 | 5.20 | 0.07 | 0.73 | 0.50 | 0.81 | 0.68 | 0.69 | -1312 |
| *Null* | 2 | 365.40 | 20.60 | 0.00 | 0 | — | — | — | — | — |

Table F. Summary information, predicted stable density (given current PIKE), and ecological benchmark density (given zero PIKE), and comparisons between most recent density and population size estimates and ecological benchmark density and population size for 73 protected areas across 21 countries. Most recent density estimates, predicted stable density, and ecological benchmark density values are represented in elephants/km^2^. PIKE estimates in bold are from empirical data while non-bold values are modeled (Table D). * indicates that an explanatory variable for a protected area is beyond the range of the original training data used to create models; estimates for these populations should be treated with caution given that extrapolating from GAMs can be problematic. PI=Prediction Interval; NP = National Park; GR = Game Reserve; GMA = Game Management Area.

| Protected area | Country | Area (km^2^) | Recent survey year | Recent density | PIKE | Mean EVI (2000-2013) | Prop. within 12km of water | Predicted stable density (95% PI) given current PIKE | Ecological benchmark density (95% PI) given zero PIKE | Recent density / ecological benchmark (%) | Recent population estimate – ecological benchmark (95% PI) |
| --- | --- | --- | --- | --- | --- | --- | --- | --- | --- | --- | --- |
| Akagera NP | Rwanda | 1020 | 2013 | 0.031 | 0.66 | 0.324 | 100.000 | 1.063 (0.514, 2.145) | 4.309 (1.284, 14.741) | 0.72 | -4363 (-15004, -1278) |
| Amboseli Ecosystem | Kenya | 6450 | 2013 | 0.21 | 0.10 | 0.195 | 78.331 | 0.477 (0.257, 0.899) | 0.585 (0.3, 1.166) | 35.80 | -2423 (-6168, -584) |
| Arli NP | Burkina | 1894 | 2013 | 0.729 | 0.85 | 0.265 | 98.963 | 0.492 (0.167, 1.332) | 2.987 (1.185, 7.542) | 24.39 | -4278 (-12905, -865) |
| Bangweulu System | Zambia | 16941 | 2008 | 0.009 | 0.72 | 0.295 | 84.027 | 0.641 (0.32, 1.206) | 2.969 (1.129, 7.815) | 0.30 | -50141 (-132235, -18971) |
| Banhine NP | Mozambique | 6051 | 2012 | 0.001 | 0.76 | 0.257 | 54.308 | 0.234 (0.098, 0.53) | 1.197 (0.544, 2.66) | 0.07 | -7236 (-16088, -3285) |
| Binder Lere Faunal Reserve | Chad | 1129 | 2013 | 0.067 | 0.77 | 0.266 | 80.029 | 0.413 (0.174, 0.905) | 2.129 (0.946, 4.787) | 3.13 | -2329 (-5329, -993) |
| Bouba Ndjida NP | Cameroon | 2115 | 2008 | 0.11 | 0.75 | 0.306 | 88.620 | 0.682 (0.331, 1.328) | 3.391 (1.197, 9.656) | 3.24 | -6939 (-20190, -2299) |
| Caprivi Region | Namibia | 17805 | 2013 | 0.548 | **0.39** | 0.274 | 60.689 | 0.72 (0.461, 1.101) | 1.643 (0.713, 3.812) | 33.34 | -19500 (-58121, -2949) |
| Charara Safari Area | Zimbabwe | 1728 | 2003 | 1.086 | 0.59 | 0.302 | 55.355 | 0.517 (0.329, 0.79) | 1.838 (0.7, 4.916) | 59.07 | -1300 (-6618, 667) |
| Chete Safari Area | Zimbabwe | 1084 | 2006 | 0.771 | 0.72 | 0.287 | 96.655 | 0.758 (0.33, 1.634) | 3.544 (1.29, 9.747) | 21.74 | -3006 (-9730, -563) |
| Chewore Safari Area | Zimbabwe | 3400 | 2010 | 1.484 | **0.39** | 0.312 | 73.170 | 1.143 (0.699, 1.854) | 2.608 (0.95, 7.245) | 56.92 | -3819 (-19587, 1817) |
| Chirisa Safari Area | Zimbabwe | 1710 | 2006 | 2.767 | 0.62 | 0.292 | 89.877 | 0.86 (0.449, 1.573) | 3.247 (1.211, 8.711) | 85.23 | -820 (-10164, 2662) |
| Chizarira NP | Zimbabwe | 1948 | 2006 | 1.474 | 0.65 | 0.293 | 82.024 | 0.701 (0.379, 1.228) | 2.83 (1.096, 7.317) | 52.06 | -2643 (-11383, 735) |
| Chobe NP | Botswana | 10550 | 2011 | 1.997 | **0.09** | 0.283 | 77.386 | 2.017 (0.94, 4.314) | 2.421 (0.999, 5.879) | 82.49 | -4472 (-40949, 10531) |
| Digya NP | Ghana | 2771 | 2006 | 0.103 | 0.64 | 0.373 | 72.373 | 0.557 (0.266, 1.184) | 2.19 (0.514, 10.039) | 4.69 | -5783 (-27533, -1139) |
| Etosha NP | Namibia | 22158 | 2011 | 0.175 | **0.00** | 0.175 | 50.385 | 0.233 (0.09, 0.621) | 0.233 (0.09, 0.621) | 74.99 | -1290 (-9899, 1863) |
| Fazao Malfakassa NP | Togo | 2153 | 2002 | 0.032 | 0.82 | 0.372 | 48.713 | 0.254 (0.124, 0.523) | 1.451 (0.34, 6.811) | 2.19 | -3056 (-14595, -663) |
| Gashaka Gumti NP | Nigeria | 5868 | 2002 | 0.003 | 0.76 | 0.388 | 86.941 | 0.514 (0.196, 1.367) | 2.59 (0.496, 14.686) | 0.13 | -15179 (-86156, -2892) |
| Gile National Reserve | Mozambique | 2840 | 2012 | 0.009 | 0.88* | 0.409* | 53.703 | 0.188 (0.059, 0.61) | 1.23 (0.19, 9.098) | 0.70 | -3469 (-25815, -516) |
| Gonarezhou NP | Zimbabwe | 4922 | 2013 | 2.054 | 0.55 | 0.274 | 79.862 | 0.719 (0.411, 1.196) | 2.324 (0.993, 5.433) | 88.40 | -1326 (-16627, 5223) |
| Gorongosa NP | Mozambique | 3675 | 2012 | 0.024 | 0.85 | 0.380 | 91.181 | 0.477 (0.19, 1.204) | 2.948 (0.604, 15.497) | 0.80 | -10748 (-56863, -2132) |
| Hurungwe Safari Area | Zimbabwe | 2888 | 2003 | 1.549 | 0.60 | 0.314 | 85.692 | 0.913 (0.531, 1.529) | 3.288 (1.13, 9.65) | 47.12 | -5022 (-23394, 1210) |
| Hwange NP | Zimbabwe | 14699 | 2007 | 2.255 | 0.25 | 0.293 | 60.560 | 1.134 (0.641, 2.001) | 1.924 (0.769, 4.87) | 117.20 | 4865 (-38441, 21848) |
| Kafue NP | Zambia | 22185 | 2011 | 0.102 | 0.60 | 0.300 | 55.570 | 0.506 (0.318, 0.78) | 1.834 (0.703, 4.874) | 5.54 | -38436 (-105873, -13333) |
| Kansonso-Busanga GMA | Zambia | 7300 | 2011 | 0.003 | 0.66 | 0.330 | 40.102 | 0.358 (0.212, 0.603) | 1.469 (0.457, 5.042) | 0.22 | -10702 (-36781, -3310) |
| Kasungu NP | Malawi | 2341 | 2005 | 0.024 | 0.76 | 0.328 | 28.652* | 0.238 (0.117, 0.487) | 1.206 (0.349, 4.599) | 1.95 | -2768 (-10711, -763) |
| Katavi-Rukwa Region | Tanzania | 12112 | 2009 | 0.539 | **0.81** | 0.341 | 49.219 | 0.298 (0.172, 0.512) | 1.683 (0.51, 5.881) | 32.04 | -13851 (-64694, 353) |
| Khaudom NP | Namibia | 3644 | 2013 | 0.63 | 0.35 | 0.246 | 85.626 | 0.845 (0.516, 1.355) | 1.779 (0.853, 3.711) | 35.43 | -4186 (-11225, -810) |
| Kigosi GR | Tanzania | 8267 | 2009 | 0.758 | 0.75 | 0.343 | 77.304 | 0.55 (0.315, 0.94) | 2.752 (0.823, 9.539) | 27.54 | -16487 (-72592, -539) |
| Kissama NP | Angola | 8597 | 2002 | 0.005 | 0.63 | 0.288 | 38.673 | 0.33 (0.172, 0.617) | 1.268 (0.466, 3.574) | 0.42 | -10857 (-30683, -3960) |
| Kizigo GR | Tanzania | 5334 | 2013 | 0.354 | 0.66 | 0.297 | 32.873 | 0.3 (0.15, 0.592) | 1.217 (0.413, 3.794) | 29.10 | -4602 (-18350, -314) |
| Kruger NP | South Africa | 18988 | 2012 | 0.844 | **0.03** | 0.254 | 97.979 | 2.349 (1.035, 5.342) | 2.513 (1.066, 5.94) | 33.60 | -31684 (-96747, -4207) |
| Limpopo NP | Mozambique | 10786 | 2010 | 0.101 | 0.74 | 0.274 | 90.762 | 0.587 (0.252, 1.275) | 2.856 (1.15, 7.071) | 3.55 | -29708 (-75171, -11313) |
| Luiana NP | Angola | 10436 | 2005 | 0.189 | 0.63 | 0.275 | 59.194 | 0.423 (0.233, 0.732) | 1.623 (0.698, 3.805) | 11.62 | -14968 (-37739, -5315) |
| Lumimba GMA | Zambia | 4371 | 2012 | 0.017 | 0.69 | 0.312 | 33.459 | 0.295 (0.154, 0.563) | 1.297 (0.42, 4.279) | 1.29 | -5598 (-18632, -1763) |
| Lupande GMA | Zambia | 4343 | 2012 | 0.115 | 0.64 | 0.324 | 28.901 | 0.31 (0.161, 0.613) | 1.214 (0.36, 4.504) | 9.49 | -4772 (-19059, -1063) |
| Mana Pools NP | Zimbabwe | 2122 | 2003 | 1.959 | 0.55 | 0.312 | 84.679 | 0.984 (0.585, 1.618) | 3.213 (1.123, 9.273) | 60.97 | -2661 (-15519, 1775) |
| Masai Mara NP | Kenya | 1766 | 2010 | 1.172 | 0.44 | 0.322 | 99.202 | 1.678 (0.81, 3.443) | 4.245 (1.284, 14.289) | 27.60 | -5428 (-23165, -198) |
| Maswa GR | Tanzania | 2878 | 2009 | 0.058 | 0.67 | 0.277 | 28.530* | 0.234 (0.101, 0.531) | 0.97 (0.336, 2.992) | 5.95 | -2626 (-8445, -801) |
| Matusadona NP | Zimbabwe | 1422 | 2006 | 1.362 | 0.64 | 0.294 | 76.805 | 0.668 (0.38, 1.108) | 2.6 (1.018, 6.659) | 52.40 | -1760 (-7532, 490) |
| Mikumi NP | Tanzania | 3256 | 2013 | 0.113 | **0.57** | 0.391 | 57.635 | 0.441 (0.162, 1.251) | 1.492 (0.288, 8.627) | 7.58 | -4491 (-27721, -571) |
| Mkomazi GR | Tanzania | 3419 | 2011 | 0.073 | 0.65 | 0.245 | 26.345* | 0.155 (0.058, 0.408) | 0.619 (0.224, 1.836) | 11.79 | -1868 (-6027, -517) |
| Mole NP | Ghana | 4494 | 2006 | 0.089 | **0.61** | 0.324 | 71.560 | 0.699 (0.455, 1.047) | 2.563 (0.88, 7.646) | 3.47 | -11120 (-33961, -3553) |
| Moremi GR | Botswana | 4890 | 2010 | 1.84 | 0.21 | 0.262 | 99.977 | 1.87 (0.916, 3.782) | 2.922 (1.168, 7.328) | 62.99 | -5288 (-26834, 3285) |
| Moyowosi GR | Tanzania | 11406 | 2009 | 0.563 | 0.59 | 0.343 | 52.697 | 0.505 (0.316, 0.813) | 1.777 (0.537, 6.225) | 31.65 | -13857 (-64582, 296) |
| Muhesi GR | Tanzania | 3771 | 2013 | 0.101 | 0.67 | 0.297 | 51.459 | 0.405 (0.235, 0.678) | 1.679 (0.643, 4.468) | 6.00 | -5950 (-16468, -2045) |
| Mulobezi GMA | Zambia | 3154 | 2011 | 0.229 | 0.58 | 0.298 | 18.145* | 0.276 (0.118, 0.676) | 0.946 (0.278, 3.639) | 24.19 | -2262 (-10755, -155) |
| Mumbwa GMA | Zambia | 3238 | 2011 | 0.133 | 0.61 | 0.317 | 55.836 | 0.527 (0.35, 0.779) | 1.933 (0.687, 5.589) | 6.87 | -5828 (-17668, -1794) |
| Munyamadzi GMA | Zambia | 3606 | 2012 | 0.537 | 0.67 | 0.333 | 92.458 | 0.89 (0.471, 1.653) | 3.726 (1.114, 12.738) | 14.40 | -11502 (-43997, -2080) |
| Murchison Falls NP | Uganda | 3878 | 2012 | 0.32 | **0.77** | 0.404 | 88.883 | 0.468 (0.146, 1.547) | 2.4 (0.38, 16.816) | 13.35 | -8066 (-63969, -230) |
| Niassa GR | Mozambique | 42298 | 2011 | 0.284 | **0.79** | 0.334 | 36.718 | 0.253 (0.135, 0.472) | 1.377 (0.41, 4.999) | 20.65 | -46213 (-199435, -5328) |
| North Luangwa NP | Zambia | 4510 | 2012 | 0.474 | 0.65 | 0.325 | 51.524 | 0.448 (0.292, 0.682) | 1.799 (0.608, 5.535) | 26.33 | -5978 (-22826, -604) |
| Nyika NP | Malawi | 3117 | 2013 | 0.028 | 0.67 | 0.361 | 54.692 | 0.413 (0.231, 0.746) | 1.711 (0.451, 6.983) | 1.64 | -5246 (-21680, -1318) |
| Pendjari NP | Benin | 2827 | 2013 | 0.432 | **0.58** | 0.275 | 88.195 | 0.581 (0.259, 1.216) | 2.74 (1.119, 6.694) | 15.78 | -6524 (-17703, -1943) |
| Quirimbas NP | Mozambique | 7913 | 2011 | 0.057 | **0.73** | 0.376 | 66.708 | 0.331 (0.156, 0.702) | 1.942 (0.446, 9.181) | 2.92 | -14917 (-72199, -3077) |
| Ruaha NP | Tanzania | 20154 | 2013 | 0.45 | 0.83 | 0.293 | 54.265 | 0.409 (0.232, 0.693) | 1.73 (0.678, 4.475) | 26.03 | -25786 (-81125, -4598) |
| Rungwa GR | Tanzania | 9734 | 2009 | 0.876 | **0.68** | 0.297 | 36.028 | 0.304 (0.155, 0.583) | 1.285 (0.448, 3.862) | 68.21 | -3976 (-29066, 4172) |
| Sapi Safari Area | Zimbabwe | 1194 | 2003 | 1.986 | **0.68** | 0.316 | 98.080 | 1.121 (0.562, 2.174) | 4.138 (1.301, 13.327) | 47.99 | -2570 (-13542, 817) |
| Selous GR | Tanzania | 47666 | 2013 | 0.226 | 0.61 | 0.358 | 65.606 | 0.619 (0.342, 1.135) | 2.1 (0.57, 8.228) | 10.75 | -89344 (-381440, -16407) |
| Serengeti NP | Tanzania | 13068 | 2009 | 0.211 | **0.57** | 0.279 | 44.090 | 0.44 (0.252, 0.75) | 1.304 (0.512, 3.389) | 16.19 | -14285 (-41521, -3934) |
| Sioma Ngwezi NP | Zambia | 4449 | 2013 | 0.031 | 0.51 | 0.302 | 32.711 | 0.38 (0.201, 0.718) | 1.242 (0.414, 3.963) | 2.48 | -5390 (-17495, -1706) |
| South Luangwa NP | Zambia | 8931 | 2012 | 0.253 | 0.56 | 0.326 | 63.735 | 0.682 (0.459, 1.001) | 2.227 (0.764, 6.696) | 11.36 | -17633 (-57545, -4559) |
| Southern NP | South Sudan | 14680 | 2007 | 0.029 | **0.55** | 0.334 | 49.350 | 0.368 (0.23, 0.588) | 1.717 (0.547, 5.662) | 1.71 | -24774 (-82693, -7602) |
| Tarangire NP | Tanzania | 2844 | 2009 | 0.805 | 0.72 | 0.268 | 94.205 | 1.034 (0.552, 1.865) | 2.818 (1.146, 6.911) | 28.55 | -5726 (-17368, -972) |
| Tsavo NP (East and West) | Kenya | 20052 | 2011 | 0.402 | **0.47** | 0.206 | 54.324 | 0.211 (0.103, 0.432) | 0.477 (0.229, 1.021) | 84.14 | -1518 (-12425, 3465) |
| Tuli Region | Botswana | 1144 | 2010 | 0.493 | **0.38** | 0.177 | 99.001 | 0.328 (0.143, 0.761) | 0.585 (0.26, 1.345) | 84.35 | -105 (-975, 266) |
| Ugalla River GR | Tanzania | 4692 | 2009 | 0.164 | 0.27 | 0.303 | 51.291 | 0.426 (0.257, 0.687) | 1.723 (0.643, 4.727) | 9.50 | -7318 (-21410, -2248) |
| W du Burkina Faso NP | Burkina | 2429 | 2013 | 0.955 | 0.66 | 0.270 | 97.676 | 0.533 (0.192, 1.368) | 3.088 (1.209, 7.895) | 30.91 | -5182 (-16858, -617) |
| W du Niger NP | Niger | 2212 | 2012 | 0.073 | 0.82 | 0.252 | 96.021 | 0.687 (0.325, 1.377) | 2.368 (1.029, 5.459) | 3.06 | -5078 (-11915, -2115) |
| Waza NP | Cameroon | 1407 | 2007 | 0.125 | **0.30** | 0.224 | 92.509 | 0.72 (0.418, 1.224) | 1.378 (0.692, 2.765) | 9.06 | -1764 (-3715, -799) |
| West Lunga NP | Zambia | 1763 | 1996 | 0.309 | 0.65 | 0.374 | 96.513 | 0.847 (0.339, 2.146) | 3.369 (0.709, 17.112) | 9.17 | -5395 (-29624, -706) |
| Yankari NP | Nigeria | 2134 | 2011 | 0.037 | **0.60** | 0.289 | 71.978 | 0.641 (0.379, 1.026) | 2.304 (0.935, 5.708) | 1.58 | -4839 (-12103, -1917) |
| Zakouma NP | Chad | 2342 | 2011 | 0.137 | **0.85** | 0.259 | 80.785 | 0.326 (0.118, 0.824) | 1.99 (0.911, 4.345) | 6.86 | -4341 (-9855, -1813) |

Supporting Information References

1. Junker J. An analysis of numerical trends in African elephant populations. M.Sc. Thesis, University of Pretoria. 2009. Available: http://www.repository.up.ac.za/handle/2263/27731.
2. Sebogo L, Barnes RFW. Strategy for the Conservation of West African Elephants. Ouagabougou; IUCN/SSC African Elephant Specialist Group; 2005.
3. Armbruster P, Lande R. A population viability analysis for African elephant (Loxodonta africana): How big should reserves be? Conserv Biol. 1993; 7: 602–610.
4. Roever CLR, Van Aarde RJ, Leggett K. Functional responses in the habitat selection of a generalist mega‐herbivore, the African savannah elephant. Ecography. 2012; 35: 972–982.
5. Pettorelli N, Vik JO, Mysterud A, Gaillard JM, Tucker CJ, Stenseth NC. Using the satellite-derived NDVI to assess ecological responses to environmental change. Trends Ecol Evol. 2005; 20: 503–510.
6. Marshal JP, Rajah A, Parrini F, Henley M, Henley SR, Erasmus BF. Scale-dependent selection of greenness by African elephants in the Kruger-private reserve transboundary region, South Africa. Eur J Wildl Res. 2011; 57: 537–548.
7. Duffy JP, Pettorelli N. Exploring the relationship between NDVI and African elephant population density in protected areas. Afr J Ecol. 2012; 50: 455–463.
8. Huete A, Didan K, Miura T, Rodriguez EP, Gao X, Ferreira LG. Overview of the radiometric and biophysical performance of the MODIS vegetation indices. Remote sens environ. 2002; 83: 195–213.
9. Teitelbaum CS, Fagan WF, Fleming CH, Dressler G, Calabrese JM, Leimgruber P, et al. How far to go? Determinants of migration distance in land mammals. Ecol Lett. 2015; 18: 545–552.
10. Hijmans RJ, Cameron SE, Parra JL, Jones PG, Jarvis A. Very high resolution interpolated climate surfaces for global land areas. [Int J Climatol. 2005; 25: 1965–1978](http://onlinelibrary.wiley.com/doi/10.1002/joc.1276/pdf).
11. Loarie SR, van Aarde RJ, Pimm SL. Fences and artificial water affect African savannah elephant movement patterns. Biol Conserve. 2009; 142: 3086–3098.
12. Illius AW, O'Connor TG. Resource heterogeneity and ungulate population dynamics. Oikos. 2000; 89: 283–294.
13. Carroll ML, Townshend JR, DiMiceli CM, Noojipady P, Sohlberg RA. A new global raster water mask at 250 m resolution. Int J Digit Earth. 2009; 2: 291–308.
14. Feng M, Sexton JO, Channan S, Townshend JR. A global, high-resolution (30-m) inland water body dataset for 2000: first results of a topographic–spectral classification algorithm. Int J Digit Earth. 2015; 9: 1–21, doi:10.1080/17538947.2015.1026420.
15. United States Geological Survey. How does Landsat 8’s 12-bit data improve data products? 2014. Available: <http://landsat.usgs.gov/L8_12_bit.php>. Accessed 01 September 2015.
16. McFeeters SK. The use of the Normalized Difference Water Index (NDWI) in the delineation of open water features. Int J Remote Sens. 1996; 17: 1425­–1432.
17. Xu H. Modification of normalised difference water index (NDWI) to enhance open water features in remotely sensed imagery. Int J Remote Sens. 2006; 27: 3025–3033.
18. Tucker CJ, Pinzon JE, Brown ME, Slayback DA, Pak EW, Mahoney R, et al. An extended AVHRR 8‐km NDVI dataset compatible with MODIS and SPOT vegetation NDVI data. Int J Remote Sens. 2005; 26: 4485–4498.
19. Key CH, Benson NC. Landscape Assessment: Remote Sensing of Severity, the Normalized Burn Ratio; and Ground Measure of Severity, the Composite Burn Index. FIREMON: Fire Effects Monitoring and Inventory System. Ogden: RMRS-GTR, USDA Forest Service, Rocky Mountain Research Station; 2006.
20. Verpoorter C, Kutser T, Tranvik L. Automated mapping of water bodies using Landsat multispectral data. Limnol Oceanogr-Meth . 2012; 10: 1037­–1050.
21. T. Tachikawa, Kaku M, Iwasaki A, Gesch DB, Oimoen MJ, Zhang Z, et al. ASTER global digital elevation model version 2-summary of validation results. NASA; 2011.
22. Horn BK. Hill shading and the reflectance map. P IEEE. 1981; 69: 14–47.
23. Owen HJF, Duncan C, Pettorelli N. Testing the water: detecting artificial water points using freely available satellite data and open source software. Remote Sensing in Ecology and Conservation. 2015; 1: 61–72.
24. Cutler DR, Edwards TC, Beard KH, Cutler A, Hess KT, Gibson J, et al. Random forests for classification in ecology. Ecology. 2007; 88: 2783–2792.
25. Gislason PO, Benediktsson JA, Sveinsson JR. Random forests for land cover classification. Pattern Recogn Letters. 2006; 27: 294–300.
26. Google Earth. Google Earth v. 7.1.2.2041. Google, Mountain View, CA. 2015. Available at: http://www.google.com/ earth. Accessed 20 November 2015.
27. Chamaillé-Jammes S, Mtare G, Makuwe E, Fritz H. African elephants adjust speed in response to surface-water constraint on foraging during the dry-season. PloS One. 2013; 8: e59164, doi:10.1371/journal.pone.0059164.
28. Polansky L, Kilian W, Wittemyer G. Elucidating the significance of spatial memory on movement decisions by African savannah elephants using state–space models. P Roy Soc Lond B Bio. 2015; 282: 20143042, doi:10.1098/rspb.2014.3042.
29. Burn RW, Underwood FM, Blanc J. Global trends and factors associated with the illegal killing of elephants: a hierarchical Bayesian analysis of carcass encounter data. PLoS One. 2011; 6: e24165, doi:10.1371/journal.pone.0024165.
30. Bhatt S, Weiss DJ, Cameron E, Bisanzio D, Mappin B, Dalrymple U, Battle KE, et al. The effect of malaria control on *Plasmodium falciparum* in Africa between 2000 and 2015. Nature. 2015; 526: 207–211.
31. Sachs J, Malaney P. The economic and social burden of malaria. Nature. 2002; 415: 680–685.
32. Trimble MJ, van Aarde RJ. Species inequality in scientific study. Conserv Biol 2010; 24: 886-890.
33. Trimble MJ, van Aarde RJ. Geographical and taxonomic biases in research on biodiversity in human-modified landscapes. Ecosphere. 2012; 3(12): 119.
34. Burnham KP, Anderson DR. Model selection and Multimodel Inference: A Practical Information-Theoretic Approach. 2^nd^ ed. New York: Springer-Verlag; 2000.
